# Analytical Impedance Model of T-II Composite-Core ECT Probe Using Truncated Region Eigenfunction Expansion

**DOI:** 10.3390/s26185756

**Published:** 2026-09-10

**Authors:** Siquan Zhang

**Affiliations:** Department of Electrical and Automation, Shanghai Maritime University, Shanghai 201306, China; sqzhang@shmtu.edu.cn

**Keywords:** eddy current testing, Truncated Region Eigenfunction Expansion, analytical model, T–II composite-core probe, multi-layer conductor, subsurface defect

## Abstract

To address the limitations of traditional analytical models in characterising multi-core coupling effects and the excessive computational cost of finite element simulations, this paper presents a high-precision analytical model for a novel T-II composite-core eddy current testing (ECT) probe using the Truncated Region Eigenfunction Expansion (TREE) method. The analytical expressions of coil impedance are derived by partitioning the solution domain into ten subdomains under an axisymmetric cylindrical coordinate system, with rigorous satisfaction of electromagnetic continuity at all material interfaces. Numerical cross-validation against 2D and 3D Finite Element Method (FEM) simulations under idealised modelling assumptions across the frequency range of 100 Hz to 10 kHz shows that the proposed TREE model yields relative errors below 2% for both coil resistance and reactance. Notably, the proposed approach requires significantly less computation time than 2D and 3D FEM. Further parametric analysis confirms that the proposed T-II composite-core probe delivers superior electromagnetic performance compared to conventional single-core probes, including intensified subsurface eddy current densities and improved magnetic field redistribution. This work overcomes the inherent limitations of single-core ECT analytical models, establishes a robust theoretical paradigm to interpret the distinctive electromagnetic field advantages of composite-core probes, and provides solid support for the structural optimisation of multi-core ECT sensors.

## 1. Introduction

Eddy current testing (ECT) is a widely adopted non-destructive testing (NDT) technique for evaluating surface and subsurface defects in multi-layer conductive components across aerospace, nuclear power, and transportation industries [1,2,3]. The inspection performance of ECT systems is fundamentally determined by the magnetic focusing capability of excitation-receiving probes. Conventional air-core probes, which are widely deployed in industrial practice, suffer from severe magnetic flux leakage, leading to weak eddy current excitation and insufficient detection sensitivity for identifying small subsurface defects [4]. To mitigate this limitation, Truncated Region Eigenfunction Expansion (TREE) analytical models have been developed for single ferrite-core probes, including I-core, E-core, T-core, and II-core geometries [5,6,7,8]. These models deliver efficient, high-precision analytical solutions for ferrite-core probes and greatly advance theoretical analysis of probe electromagnetic behaviour.

Since the inception of the TREE method for classic eddy current boundary value problems [9,10], researchers have built up a mature theoretical framework for single ferrite-core ECT probes. Significant progress has been achieved by researchers including Theodoulidis et al. [10], Lu et al. [5], Tytko and Dziczkowski [7,11], and Sakkaki et al. [12], who extended TREE-based modelling to diverse single-core geometries such as the I-core, E-core, and cup-core. These investigations established a solid theoretical foundation for analytical modelling of single-core ECT probes. Further investigations on ferrite-core transceiver coils and novel T-cored sensors have enriched the single-core theoretical system, which can reliably predict eddy current responses under conventional test conditions [8,13]. Overall, TREE-based modelling techniques are well established for diverse single-ferrite probe structures.

Despite the mature theoretical framework established for single-core probes, existing analytical solutions remain largely confined to homogeneous geometries. Although a limited number of studies have investigated dual-ferrite arrangements [6], these works primarily focus on shield-oriented configurations—such as the shielded dual-core probe by Vasić et al. combining an inner I-core and an outer magnetic shield. While such research addresses magnetic shielding performance, it overlooks the field modulation advantages of composite cores, specifically subsurface eddy current enhancement and magnetic field redistribution, which are vital for improving defect detectability [6,14,15,16]. Consequently, current frameworks lack the flexibility to adaptively tune eddy current patterns for complex multi-layer inspections.

**Figure 1:**
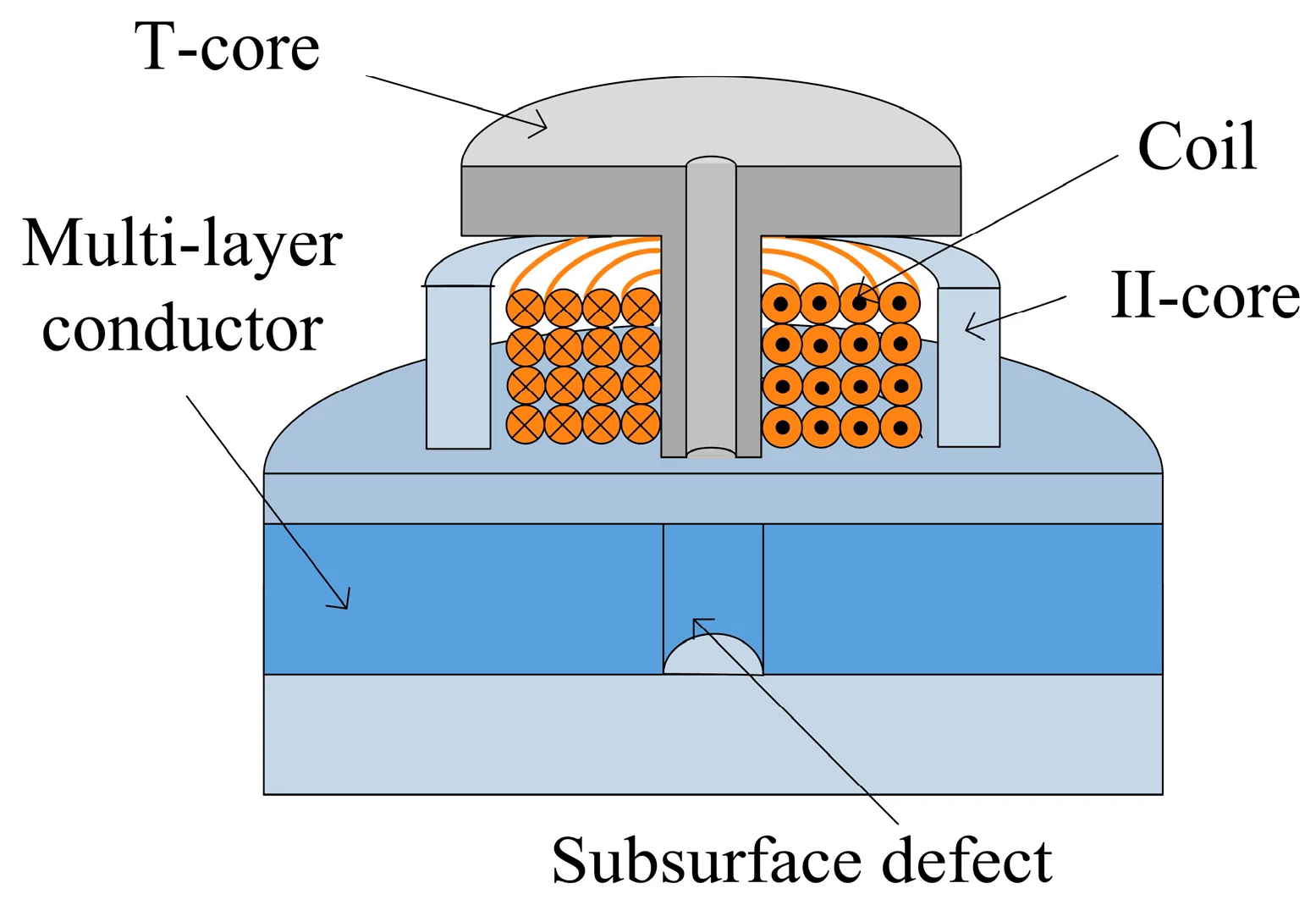
Cross-section of a composite-core probe located above layered conductors with a subsurface defect.

This limitation stems from the absence of dedicated boundary matching algorithms capable of handling the intricate electromagnetic coupling in custom-designed composite probes. Unlike single-core counterparts, composite geometries partition the solution domain into significantly more radial subdomains, rendering conventional continuity equations insufficient. This challenge is particularly prominent in the T-II composite-core probe investigated herein. As illustrated in Figure 1, its layout—comprising two ferrite cores with distinct permeabilities and multiple radial air gaps—creates ten independent electromagnetic subdomains. To the best of our knowledge, rigorous analytical impedance expressions for such T-II composite-core probes have not yet been reported, creating a distinct theoretical gap that this work aims to fill.

Beyond analytical model limitations, Finite Element Method (FEM) simulation is widely adopted as a validation benchmark within NDT research [17,18,19,20]. FEM can accurately compute magnetic-core–conductor electromagnetic coupling but suffers from low computational throughput, which impedes high-throughput parametric scanning and structural optimisation of probes. Moreover, most earlier analytical validations rely solely on 2D FEM outputs and overlook approximation errors stemming from ideal axisymmetric simplifications. Systematic cross-validation against high-fidelity 3D FEM benchmarks alongside eigenfunction series convergence analysis is still rare for multi-core composite probes, lowering the dependability and practical value of existing analytical outcomes. Furthermore, the quantitative interplay between subsurface defect detection sensitivity and the geometric parameters of composite cores, along with their resultant magnetic field distributions, remains poorly understood. This unresolved knowledge gap hinders optimised high-sensitivity ECT probe design for buried flaw inspection and impedes real-world engineering deployment of composite-core sensing hardware [21,22,23].

Recent years have witnessed multiple hardware-driven approaches for boosting subsurface detection performance of eddy current probes. For instance, Sardellitti et al. proposed an optimised pot-cored probe to enhance corrosion defect characterisation, demonstrating that specially designed magnetic-core layouts can effectively reshape magnetic field distribution and improve inspection capability [4,14]. Inspired by such advances, this work explores an alternative composite-core design route based on combined T-core and II-core ferrite segments.

The T-II composite-core geometry is deliberately selected for the following reasons. Unlike fixed structure single-ferrite probes (I-core, E-core, T-core, or II-core), this assembled dual-core layout provides flexible degrees of freedom to adjust the radial and axial positions of two dissimilar ferrite components. Such structural reconfigurability enables tunable magnetic flux focusing and redistribution, which is highly desirable for strengthening the excitation of subsurface eddy currents. This advantageous property cannot be readily achieved by conventional monolithic core structures, and motivates the present investigation of the T-II composite-core probe.

To fill the abovementioned theoretical and technical gaps, this work constructs a complete TREE-based analytical modelling framework for T-II composite-core ECT probes positioned above multi-layer conductors containing subsurface defects. Within an axisymmetric cylindrical coordinate system, the full detection domain is partitioned into ten electromagnetic subdomains matching the composite probe geometry. By enforcing rigorous continuity conditions of radial magnetic flux density *B*_r_ and axial magnetic field intensity *H*_z_ at all subdomain interfaces, analytical impedance solutions are derived for the T-II dual-core probe, fully accounting for magnetic interactions between the T-core and the II-core with different magnetic permeabilities. The proposed analytical model provides a rigorous theoretical basis for electromagnetic analysis and performance evaluation of composite dual-core ECT probes, further facilitating structural optimisation and detection performance improvement of high-sensitivity ECT sensors [24,25,26].

The remainder of this paper is organised as follows. Section 2 describes geometric features of the T-II composite-core probe, governing electromagnetic equations and the full TREE eigenfunction expansion derivation for the ten segmented subdomains. Section 3 explains the numerical solution workflow of the analytical model, presents convergence analysis for determining optimal calculation parameters, and introduces the FEM verification setup. Section 4 compares normalised impedance variations for four typical probe configurations (T-II composite-core, single T-core, single II-core, and air core) across the frequency range of 100 Hz to 10 kHz to further verify model robustness. It also discusses parametric study results to reveal the magnetic field tuning mechanism governed by composite-core geometric parameters, providing targeted structural optimisation guidance for high-sensitivity ECT inspection. Section 5 summarises the core conclusions, discusses model limitations, and outlines future research directions, including experimental validation and extended engineering applicability. Appendix A contains supplementary matrix equations and integral formulas to ensure the reproducibility and completeness of the theoretical derivation.

## 2. Analytical Modelling

**Figure 2:**
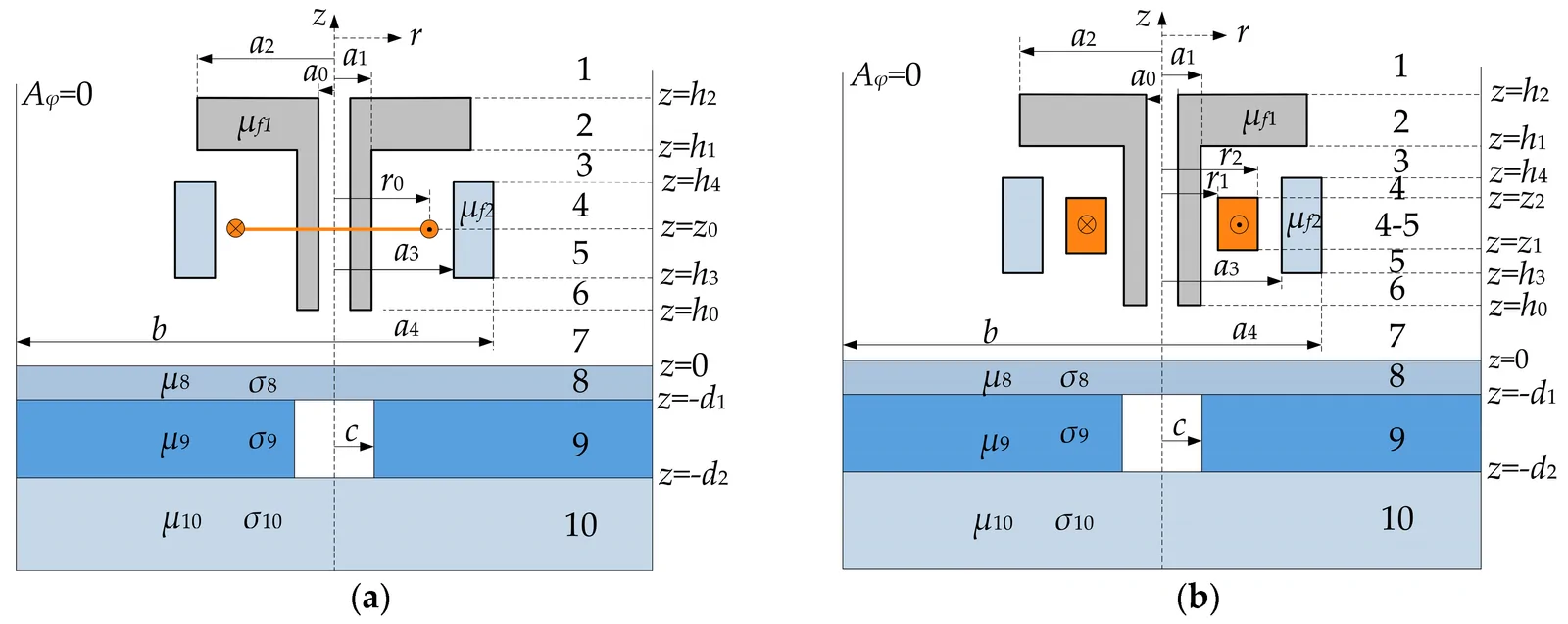
Axially symmetric model of composite-core probe above layered conductors containing a subsurface defect: (**a**) single-turn coil configuration; (**b**) multi-turn coil configuration, where the orange box denotes the multi-turn excitation coil region. The gray regions represent ferromagnetic cores, the light-blue and blue regions denote conductive layered media, and the white gap represents the subsurface defect.

The actual detection scenario (Figure 1) is simplified into the analytical model shown in Figure 2 to facilitate theoretical analysis. The axisymmetric electromagnetic boundary value problem is solved via the TREE method, where the magnetic vector potential is expanded using first-order Bessel functions of the first and second kind for cylindrical geometries [9,10].

The overall derivation follows a well-established TREE workflow. First, the complete computational domain is divided into multiple independent electromagnetic subdomains according to the complex geometric layout of the T-II composite-core probe. Second, magnetic vector potential solutions for each subdomain are constructed using Bessel function series expansions, with corresponding discrete radial eigenvalues solved for every distinct subdomain type. Third, electromagnetic continuity constraints for magnetic flux density and magnetic field intensity are enforced across all internal interfaces to assemble the system of field coefficients. Finally, the coil impedance expression is obtained by integrating the solved magnetic vector potential over the physical cross-section of the excitation winding.

The analytical model adopts an *r-z* coordinate system, which is well suited to describe the axial symmetry of the coil probe and multi-layer conductive structures. Specifically, this scenario involves a T-core and an II-core coil probe with permeabilities of *μ_f_*_1_ and *μ_f_*_2_, respectively. The coil is located in the region enclosed by the T-core and the II-core. An alternating excitation current fed into the coil induces eddy currents inside the layered conductors, and subsurface hidden defects can be characterised by extracting variations in coil impedance. The initial configuration, depicted in Figure 2a, features a single-turn coil. In Figure 2b, the setup remains identical with the exception of the coil, which is replaced by a multi-turn variant. This configuration represents the practical non-contact composite-core probe. The probe is located above a non-magnetic conducting half-space involving three layers of varying thicknesses, with conductivities of *σ*_8_, *σ*_9_, and *σ*_10_ corresponding to Regions 8, 9, and 10, respectively. The plane *z* = 0 coincides with the upper surface of the first-layer conductor. A finite cylindrical calculation domain with outer radial truncation radius *r* = *b* covers the full axial range of the probe–conductor system, and the TREE analytical model is solved within this bounded domain.

To validate the proposed multi-subdomain analytical framework, an axisymmetric annular hole is introduced into the second layer of the multi-layer conductive specimen as a canonical benchmark defect. This rotationally symmetric geometry serves as a well-defined test case for verifying the rigorous boundary matching across the ten electromagnetic subdomains. It is worth noting that while this idealised axisymmetric defect cannot fully represent general non-axisymmetric localised flaws (such as finite cracks, pits, or irregular corrosion regions) encountered in practical inspections, it provides an exact and mathematically tractable foundation for establishing the composite-core impedance model. Extensions to arbitrary local defect scenarios will be addressed in future work.

According to the problem geometry in Figure 2a, ten regions are formed. These ten axial regions are divided by multiple fixed *z* coordinate planes according to the axial arrangement of the ferrite cores, excitation coil and layered conductive specimen. With the first-order Bessel functions *J*_1_ and *Y*_1_, the discrete eigenvalues *q_i_*, *m_i_*, *p_i_*, *g_i_*, and *u_i_* are calculated as defined below, where each variable corresponds to the discrete radial eigenvalue of its respective domain [9,10].

Regions 1, 7, 8, and 10 consist solely of air or a single conductor, so the eigenvalues *q_i_* for these regions can be obtained by calculating the positive real roots of the following equation:

(1) $$J_{1}(q_{i}b)=0\begin{array}{cc} & i=0,1,2\ldots ,N_{s} \end{array}$$

where *N_s_* denotes the total truncation order of discrete radial eigenvalue *q_i_* adopted in the TREE method.

Region 2 is split into three subdomains. Subdomain I (0 ≤ *r* ≤ *a*_0_) and Subdomain III (*a*_2_ ≤ *r* ≤ *b*) consist of air, while Subdomain II (*a*_0_ ≤ *r* ≤ *a*_2_) contains the T-core.

The azimuthal magnetic vector potential ***A****_Φ_* in each subdomain is expressed in terms of the first-order Bessel functions of the first and second kind (*J*_1_ and *Y*_1_) as follows:

(2) $$A_{I}=A_{E}J_{1}(m_{i}r)\begin{array}{cc} & 0\leq r\leq a_{0} \end{array}$$

(3) $$A_{II}=A_{E}B_{2F}J_{1}(m_{i}r)+A_{E}C_{2F}Y_{1}(m_{i}r)\begin{array}{ccc} & a_{0}\leq r\leq a_{2} & \end{array}$$

(4) $$A_{\mathrm{III}}=A_{E}B_{3F}J_{1}(m_{i}r)+A_{E}C_{3F}Y_{1}(m_{i}r)\begin{array}{ccc} & a_{2}\leq r\leq b & \end{array}$$

The continuity relations of *B_r_* and *H_z_* hold for all radial material interfaces, as no free surface current exists on cylindrical boundaries at *r* = constant. To satisfy these two continuity conditions at the cylindrical air-gap interface of the T-core (*r* = *a*_0_), an auxiliary function is introduced and defined as:

(5) $$R_{n}(m_{i}r)=B_{2F}J_{n}(m_{i}r)+C_{2F}Y_{n}(m_{i}r),\begin{array}{cc} & n=0,1 \end{array}$$

Direct application of the field continuity conditions yields the coefficient expressions below:

(6) $$C_{2F}=\frac{\pi m_{i}a_{0}}{2}J_{0}(m_{i}a_{0})J_{1}(m_{i}a_{0})(\mu_{f1}-1)$$

(7) $$B_{2F}=\frac{\pi m_{i}a_{0}}{2}[J_{1}(m_{i}a_{0})Y_{0}(m_{i}a_{0})-J_{0}(m_{i}a_{0})Y_{1}(m_{i}a_{0})\mu_{f1}]$$

Similarly, imposing the continuity of *B_r_* and *H_z_* across the interface *r* = *a*_2_ gives the following relations:

(8) $$C_{3F}=\frac{\pi m_{i}a_{2}}{2}[\frac{J_{1}(m_{i}a_{2})R_{0}(m_{i}a_{2})}{\mu_{f1}}-J_{0}(m_{i}a_{2})R_{1}(m_{i}a_{2})]$$

(9) $$B_{3F}=\frac{\pi m_{i}a_{2}}{2}[Y_{0}(m_{i}a_{2})R_{1}(m_{i}a_{2})-\frac{Y_{1}(m_{i}a_{2})R_{0}(m_{i}a_{2})}{\mu_{f1}}]$$

The discrete eigenvalues *m_i_* correspond to all positive real roots of Equation (10), namely:

(10) $$R_{1}^{\prime }(m_{i}b)=0\begin{array}{ccc} & & i=0,1,2\ldots ,N_{s} \end{array}$$

where the auxiliary function $R_{1}^{\prime }(m_{i}r)$ is expressed as:

(11) $$R_{1}^{\prime }(m_{i}r)=B_{3F}J_{1}(m_{i}r)+C_{3F}Y_{1}(m_{i}r)$$

Regions 3 and 6 are each partitioned into three distinct subdomains. Subdomain I (0 ≤ *r* ≤ *a*_0_) and Subdomain III (*a*_1_ ≤ *r* ≤ *b*) are filled with air, whereas Subdomain II (*a*_0_ ≤ *r* ≤ *a*_1_) accommodates the ferrite magnetic core.

The azimuthal magnetic vector potential $A_{\varphi }^{\prime }$ within each subdomain can be expanded using first-order Bessel functions of the first and second kind (*J*_1_ and *Y*_1_), as presented below:

(12) $$A_{I}^{\prime }=A_{E}J_{1}(p_{i}r)\begin{array}{ccc} & 0\leq r\leq a_{0} & \end{array}$$

(13) $$A_{II}^{\prime }=A_{E}B_{2F}^{\prime }J_{1}(p_{i}r)+A_{E}C_{2F}^{\prime }Y_{1}(p_{i}r)\begin{array}{ccc} & a_{0}\leq r\leq a_{1} & \end{array}$$

(14) $$A_{III}^{\prime }=A_{E}B_{3F}^{\prime }J_{1}(p_{i}r)+A_{E}C_{3F}^{\prime }Y_{1}(p_{i}r)\begin{array}{ccc} & a_{1}\leq r\leq b & \end{array}$$

To enforce continuity of the radial magnetic flux density *B_r_* and axial magnetic field intensity *H_z_* across the interface *r* = *a*_0_, an auxiliary function $L_{n}(p_{i}r)$ is first defined as:

(15) $$L_{n}(p_{i}r)=B_{2F}^{\prime }J_{n}(p_{i}r)+C_{2F}^{\prime }Y_{n}(p_{i}r),\begin{array}{cc} & n=0,1 \end{array}$$

Direct substitution of these continuity conditions yields the coefficient expressions given below:

(16) $$C_{2F}^{\prime }=\frac{\pi p_{i}a_{0}}{2}J_{0}(p_{i}a_{0})J_{1}(p_{i}a_{0})(\mu_{f1}-1)$$

(17) $$B_{2F}^{\prime }=\frac{\pi p_{i}a_{0}}{2}[J_{1}(p_{i}a_{0})Y_{0}(p_{i}a_{0})-J_{0}(p_{i}a_{0})Y_{1}(p_{i}a_{0})\mu_{f1}]$$

Similarly, enforcing continuity for *B_r_* and *H_z_* across the boundary *r* = *a*_1_ gives the relations:

(18) $$C_{3F}^{\prime }=\frac{\pi p_{i}a_{1}}{2}[\frac{J_{1}(p_{i}a_{1})L_{0}(p_{i}a_{1})}{\mu_{f1}}-J_{0}(p_{i}a_{1})L_{1}(p_{i}a_{1})]$$

(19) $$B_{3F}^{\prime }=\frac{\pi p_{i}a_{1}}{2}[Y_{0}(p_{i}a_{1})L_{1}(p_{i}a_{1})-\frac{Y_{1}(p_{i}a_{1})L_{0}(p_{i}a_{1})}{\mu_{f1}}]$$

The discrete eigenvalues *p_i_* correspond to all positive real roots obtained by solving the characteristic equation:

(20) $$L_{1}^{\prime }(p_{i}b)=0\begin{array}{ccc} & & i=0,1,2\ldots ,N_{s} \end{array}$$

where the auxiliary function $L_{1}^{\prime }(p_{i}r)$ is defined as:

(21) $$L_{1}^{\prime }(p_{i}r)=B_{3F}^{\prime }J_{1}(p_{i}r)+C_{3F}^{\prime }Y_{1}(p_{i}r)$$

Regions 4 and 5 are each partitioned into five separate subdomains: Subdomain I (0 ≤ *r* ≤ *a*_0_), Subdomain II (*a*_0_ ≤ *r* ≤ *a*_1_), Subdomain III (*a*_1_ ≤ *r* ≤ *a*_3_), Subdomain IV (*a*_3_ ≤ *r* ≤ *a*_4_), and Subdomain V (*a*_4_ ≤ *r* ≤ *b*). Subdomains I, III, and V are air-filled regions, while Subdomains II and IV consist of ferrite material corresponding to the T-core and II-core, respectively.

Applying continuity constraints for *B_r_* and *H_z_* across the interface *r* = *a*_0_ yields the following coefficient expressions:

(22) $$C_{2F}^{\prime \prime }=\frac{\pi g_{i}a_{0}}{2}J_{0}(g_{i}a_{0})J_{1}(g_{i}a_{0})(\mu_{f1}-1)$$

(23) $$B_{2F}^{\prime \prime }=\frac{\pi g_{i}a_{0}}{2}[J_{1}(g_{i}a_{0})Y_{0}(g_{i}a_{0})-\mu_{f1}J_{0}(g_{i}a_{0})Y_{1}(g_{i}a_{0})]$$

Implementing identical continuity constraints for *B_r_* and *H_z_* across the boundary *r* = *a*_1_ yields the relations below:

(24) $$C_{3F}^{\prime \prime }=\frac{\pi g_{i}a_{1}}{2}[\frac{1}{\mu_{f1}}J_{1}(g_{i}a_{1})X_{0}(g_{i}a_{1})-J_{0}(g_{i}a_{1})X_{1}(g_{i}a_{1})]$$

(25) $$B_{3F}^{\prime \prime }=\frac{\pi g_{i}a_{1}}{2}[Y_{0}(g_{i}a_{1})X_{1}(g_{i}a_{1})-\frac{1}{\mu_{f1}}Y_{1}(g_{i}a_{1})X_{0}(g_{i}a_{1})]$$

where the auxiliary function $X_{n}^{\prime }(g_{i}r)$ is defined as:

(26) $$X_{n}^{\prime }(g_{i}r)=B_{3F}^{\prime \prime }J_{n}(g_{i}r)+C_{3F}^{\prime \prime }Y_{n}(g_{i}r),\begin{array}{cc} & n=0,1 \end{array}$$

Likewise, invoking the continuity requirements of *B_r_* and *H_z_* across the boundary *r* = *a*_3_ results in the formula shown below:

(27) $$C_{4F}^{\prime \prime }=\frac{\pi g_{i}a_{3}}{2}[\mu_{f2}J_{1}(g_{i}a_{3})X_{0}^{\prime }(g_{i}a_{3})-J_{0}(g_{i}a_{3})X_{1}^{\prime }(g_{i}a_{3})]$$

(28) $$B_{4F}^{\prime \prime }=\frac{\pi g_{i}a_{3}}{2}[Y_{0}(g_{i}a_{3})X_{1}^{\prime }(g_{i}a_{3})-\mu_{f2}Y_{1}(g_{i}a_{3})X_{0}^{\prime }(g_{i}a_{3})]$$

where the auxiliary function $X_{1}^{\prime \prime }(g_{i}r)$ is defined as:

(29) $$X_{1}^{\prime \prime }(g_{i}r)=B_{4F}^{\prime \prime }J_{1}(g_{i}r)+C_{4F}^{\prime \prime }Y_{1}(g_{i}r)$$

Enforcing continuity constraints for *B_r_* and *H_z_* across the boundary *r* = *a*_4_ yields the relations below:

(30) $$C_{5F}^{\prime \prime }=\frac{\pi g_{i}a_{4}}{2}[\frac{1}{\mu_{f2}}J_{1}(g_{i}a_{4})X_{0}^{\prime \prime }(g_{i}a_{4})-J_{0}(g_{i}a_{4})X_{1}^{\prime \prime }(g_{i}a_{4})]$$

(31) $$B_{5F}^{\prime \prime }=\frac{\pi g_{i}a_{4}}{2}[Y_{0}(g_{i}a_{4})X_{1}^{\prime \prime }(g_{i}a_{4})-\frac{1}{\mu_{f2}}Y_{1}(g_{i}a_{4})X_{0}^{\prime \prime }(g_{i}a_{4})]$$

The discrete eigenvalues *g_i_* correspond to all positive real roots of the characteristic equation:

(32) $$X_{1}^{\prime \prime \prime }(g_{i}b)=0\begin{array}{ccc} & & i=0,1,2\ldots ,N_{s} \end{array}$$

where the auxiliary function $X_{1}^{\prime \prime \prime }(g_{i}r)$ is expressed as:

(33) $$X_{1}^{\prime \prime \prime }(g_{i}r)=B_{5F}^{\prime \prime }J_{1}(g_{i}r)+C_{5F}^{\prime \prime }Y_{1}(g_{i}r)$$

Region 9 consists of two separate subdomains: an air-filled domain (0 ≤ *r* ≤ *c*) and a conductive domain (*c* ≤ *r* ≤ *b*). The azimuthal magnetic vector potential within each subdomain takes the following general form:

(34) $$A_{9a}=A_{E}J_{1}(u_{i}r)F_{1}(v_{i}c)\begin{array}{ccc} & 0\leq r\leq c & \end{array}$$

(35) $$A_{9c}=A_{E}J_{1}(u_{i}c)F_{1}(v_{i}r)\begin{array}{ccc} & c\leq r\leq b & \end{array}$$

where the auxiliary function $F_{1}(v_{i}r)$ is defined as:

(36) $$F_{1}(v_{i}r)=J_{1}(v_{i}r)Y_{1}(v_{i}b)-J_{1}(v_{i}b)Y_{1}(v_{i}r)$$

where *J*_1_ and *Y*_1_ represent the first-order Bessel functions of the first and second kind, respectively.

By applying the continuity of azimuthal magnetic vector potential ***A****_Φ_* at the radial material interface *r* = *c*, the following identity is obtained:

(37) $$u_{i}F_{1}(v_{i}c)J_{0}(u_{i}c)=\frac{1}{\mu_{9}}v_{i}F_{0}(v_{i}c)J_{1}(u_{i}c)$$

Equation (37) can be solved to extract the eigenvalues *u_i_* and their corresponding *v_i_*, which satisfy the following dispersion relation:

(38) $$u_{i}=\sqrt{v_{i}^{2}+j\omega \sigma_{9}\mu_{0}\mu_{9}}$$

Via separation of variables, the azimuthal magnetic vector potential ***A****_Φ_* for every domain of the geometry shown in Figure 2a can be written in piecewise matrix form, as indicated below:

(39) $$A_{1}(r,z)=J_{1}(q^{T}r)q^{-1}e^{-qz}C_{1}$$

(40) $$A_{2}(r,z)=\left\{\begin{array}{c} J_{1}(m^{T}r)\\ R_{1}(m^{T}r)\\ R_{1}^{\prime }(m^{T}r) \end{array}\right.m^{-1}(e^{-mz}C_{2}-e^{mz}B_{2})\begin{array}{ccc} & 0\leq r\leq a_{0} & \\ & a_{0}\leq r\leq a_{2} & \\ & a_{2}\leq r\leq b & \end{array}$$

(41) $$A_{3}(r,z)=\left\{\begin{array}{c} J_{1}(p^{T}r)\\ L_{1}(p^{T}r)\\ L_{1}^{\prime }(p^{T}r) \end{array}\right.p^{-1}(e^{-pz}C_{3}-e^{pz}B_{3})\begin{array}{ccc} & 0\leq r\leq a_{0} & \\ & a_{0}\leq r\leq a_{1} & \\ & a_{1}\leq r\leq b & \end{array}$$

(42) $$A_{4}(r,z)=\left\{\begin{array}{c} J_{1}(g^{T}r)\\ X_{1}(g^{T}r)\\ X_{1}^{\prime }(g^{T}r)\\ X_{1}^{\prime \prime }(g^{T}r)\\ X_{1}^{\prime \prime \prime }(g^{T}r) \end{array}\right.g^{-1}(e^{-gz}C_{4}-e^{gz}B_{4})\;\;\begin{array}{c} 0\leq r\leq a_{0}\\ a_{0}\leq r\leq a_{1}\\ a_{1}\leq r\leq a_{3}\\ a_{3}\leq r\leq a_{4}\\ a_{4}\leq r\leq b \end{array}$$

(43) $$A_{5}(r,z)=\left\{\begin{array}{c} J_{1}(g^{T}r)\\ X_{1}(g^{T}r)\\ X_{1}^{\prime }(g^{T}r)\\ X_{1}^{\prime \prime }(g^{T}r)\\ X_{1}^{\prime \prime \prime }(g^{T}r) \end{array}\right.g^{-1}(e^{-gz}C_{5}-e^{gz}B_{5})\;\;\;\;\begin{array}{c} 0\leq r\leq a_{0}\\ a_{0}\leq r\leq a_{1}\\ a_{1}\leq r\leq a_{3}\\ a_{3}\leq r\leq a_{4}\\ a_{4}\leq r\leq b \end{array}$$

(44) $$A_{6}(r,z)=\left\{\begin{array}{c} J_{1}(p^{T}r)\\ L_{1}(p^{T}r)\\ L_{1}^{\prime }(p^{T}r) \end{array}\right.p^{-1}(e^{-pz}C_{6}-e^{pz}B_{6})\begin{array}{ccc} & 0\leq r\leq a_{0} & \\ & a_{0}\leq r\leq a_{1} & \\ & a_{1}\leq r\leq b & \end{array}$$

(45) $$A_{7}(r,z)=J_{1}(q^{T}r)q^{-1}(e^{-qz}C_{7}-e^{qz}B_{7})$$

(46) $$A_{8}(r,z)=J_{1}(q^{T}r)s_{8}^{-1}(e^{-s_{8}z}C_{8}-e^{s_{8}z}B_{8})$$

(47) $$A_{9}=\left\{\begin{array}{c} J_{1}(u^{T}r)F_{1}(vc)\\ F_{1}(v^{T}r)J_{1}(uc) \end{array}\right.u^{-1}(e^{-uz}C_{9}-e^{uz}B_{9})\begin{array}{ccc} & \begin{array}{c} 0\leq r\leq c\\ c\leq r\leq b \end{array} & \end{array}$$

(48) $$A_{10}(r,z)=-J_{1}(q^{T}r)s_{10}^{-1}e^{s_{10}z}B_{10}$$

The parameters **s**_8_ and **s**_10_ are defined via the following expressions:

(49) $$s_{8}=\sqrt{q^{2}+j\omega \mu_{8}\mu_{0}\sigma_{8}}$$

(50) $$s_{10}=\sqrt{q^{2}+j\omega \mu_{10}\mu_{0}\sigma_{10}}$$

where *J*_1_ and *Y*_1_ represent the first-order Bessel functions of the first and second kinds, respectively. It should be noted that $J_{1}(q^{T}r)$, $J_{1}(m^{T}r)$, $R_{1}(m^{T}r)$, $R_{1}^{\prime }(m^{T}r)$, $J_{1}(p^{T}r)$, $L_{1}(p^{T}r)$, $L_{1}^{\prime }(p^{T}r)$, $J_{1}(g^{T}r)$, $X_{1}(g^{T}r)$, $X_{1}^{\prime }(g^{T}r)$, $X_{1}^{\prime \prime }(g^{T}r)$, $X_{1}^{\prime \prime \prime }(g^{T}r)$, $J_{1}(u^{T}r)$, and $F_{1}(v^{T}r)$ all take the form of row vectors. The inverse matrices $q^{-1}$, $m^{-1}$, $p^{-1}$, $g^{-1}$, $u^{-1}$, alongside the exponential matrices $e^{\pm qz}$, $e^{\pm mz}$, $e^{\pm pz}$, $e^{\pm gz}$, $e^{\pm uz}$, $e^{\pm s_{8}z}$, $e^{s_{10}z}$ are diagonal matrices. The column vectors **C***_i_* and **B***_i_* denote undetermined field coefficients corresponding to the *i*-th axial region and each radial discrete eigenvalue.

Equations (39)–(48) are used to calculate the azimuthal magnetic vector potential for each of the ten axial regions partitioned along the *z*-axis under excitation from the single-turn coil (shown in Figure 2a). The unknown coefficient vectors **C***_i_* and **B***_i_* are determined by combining this set of field expansion equations with interfacial continuity constraints imposed on the boundaries between the ten axial regions. For most axial interfaces at fixed *z* without surface excitation current, the governing continuity relations involve the normal magnetic flux density *B_z_* and tangential magnetic field strength *H_r_*. An exception applies to the axial boundary between Region 4 and Region 5 where the excitation coil is located; surface current density will introduce a jump in *H*_r_.

For Region 4 (*a*_1_ ≤ *r* ≤ *a*_3_, the domain situated above the single-turn coil) and Region 5 (sharing the same radial span *a*_1_ ≤ *r* ≤ *a*_3_, positioned beneath the coil as shown in Figure 2a), their respective azimuthal magnetic vector potentials are derived independently and given below:

(51) $$\begin{array}{l} A_{4}(r,z)=X_{1}^{\prime }(gr)g^{-1}(e^{-gz}C_{4}-e^{gz}B_{4})\\ =\frac{1}{2}\mu_{0}ID_{ji}^{-1}r_{0}X_{1}^{\prime }(gr_{0})g^{-1}X_{1}^{\prime }(gr)\\ \left.\cdot \left\{\left[e^{-gz}C_{510}-e^{gz}B_{510}]\frac{(\beta_{1}+\beta_{3}-\lambda_{1}-\lambda_{3})e^{gz_{0}}+(\beta_{2}+\beta_{4}-\lambda_{2}-\lambda_{4})e^{-gz_{0}}}{(\lambda_{1}+\lambda_{3}-\beta_{1}-\beta_{3})C_{510}+(\lambda_{2}+\lambda_{4}-\beta_{2}-\beta_{4})B_{510}}+[e^{g(z_{0}-z)}-e^{g(z-z_{0})}\right]\right.\right\} \end{array}$$

(52) $$\begin{array}{l} A_{5}(r,z)=X_{1}^{\prime }(gr)g^{-1}(e^{-gz}C_{5}-e^{gz}B_{5})\\ =\frac{1}{2}\mu_{0}ID_{ji}^{-1}r_{0}X_{1}^{\prime }(g_{j}r_{0})X_{1}^{\prime }(gr)g^{-1}(e^{-gz}C_{510}-e^{gz}B_{510})\\ \cdot \frac{(\beta_{1}+\beta_{3}-\lambda_{1}-\lambda_{3})e^{g_{i}z_{0}}+(\beta_{2}+\beta_{4}-\lambda_{2}-\lambda_{4})e^{-g_{i}z_{0}}}{(\lambda_{1}+\lambda_{3}-\beta_{1}-\beta_{3})C_{510}+(\lambda_{2}+\lambda_{4}-\beta_{2}-\beta_{4})B_{510}} \end{array}$$

All auxiliary parameters appearing in Equations (51) and (52), including C_510_, λ*_i_*, and β*_i_*, are defined in Appendix A.

By integrating the single-turn coil solution over the coil cross-section, the azimuthal magnetic vector potentials for the upper and lower air subdomains can be derived as presented below:

(53) $$A(r,z)=\int_{r_{1}}^{r_{2}}\int_{z_{1}}^{z_{2}}A_{filamentary}(r,z,r_{0},z_{0})dr_{0}dz_{0}$$

(54) $$\begin{array}{l} A_{4}^{coil}=\frac{\mu I}{2}D_{ji}^{-1}g_{i}^{-1}X_{1}^{\prime }(g_{i}r)[\frac{1}{g_{j}^{3}}\int_{g_{j}r_{1}}^{g_{j}r_{2}}g_{j}r_{0}X_{1}^{\prime }(g_{j}r_{0})d(g_{j}r_{0})]\\ \cdot \left\{\left(e^{-gz}C_{510}-e^{gz}B_{510})\frac{\left(\beta_{1}+\beta_{3}-\lambda_{1}-\lambda_{3})(e^{g_{i}z_{2}}-e^{g_{i}z_{1}})+(\beta_{2}+\beta_{4}-\lambda_{2}-\lambda_{4})(e^{-g_{i}z_{1}}-e^{-g_{i}z_{2}}\right)}{(\lambda_{1}+\lambda_{3}-\beta_{1}-\beta_{3})C_{510}+(\lambda_{2}+\lambda_{4}-\beta_{2}-\beta_{4})B_{510}}+[e^{g_{i}(z_{2}-z)}-e^{g_{i}(z_{1}-z)}-e^{g_{i}(z-z_{1})}+e^{g_{i}(z-z_{2})}\right]\right\} \end{array}$$

(55) $$\begin{array}{l} A_{5}^{coil}=\frac{\mu I}{2}D_{ji}^{-1}g_{i}^{-1}X_{1}^{\prime }(g_{i}r)[\frac{1}{g_{j}^{3}}\int_{g_{j}r_{1}}^{g_{j}r_{2}}g_{j}r_{0}X_{1}^{\prime }(g_{j}r_{0})d(g_{j}r_{0})](e^{-gz}C_{510}-e^{gz}B_{510})\\ \cdot \frac{\left(\beta_{1}+\beta_{3}-\lambda_{1}-\lambda_{3})(e^{g_{i}z_{2}}-e^{g_{i}z_{1}})+(\beta_{2}+\beta_{4}-\lambda_{2}-\lambda_{4})(e^{-g_{i}z_{1}}-e^{-g_{i}z_{2}}\right)}{(\lambda_{1}+\lambda_{3}-\beta_{1}-\beta_{3})C_{510}+(\lambda_{2}+\lambda_{4}-\beta_{2}-\beta_{4})B_{510}} \end{array}$$

The azimuthal magnetic vector potential $A_{4-5}^{coil}$ within the domain between Region 4 and Region 5 is computed by substituting *z* for *z*_2_ in $A_{4}^{coil}$ and substituting *z* for *z*_1_ in $A_{5}^{coil}$, followed by summing the two modified expressions, as shown below:

(56) $$\begin{array}{l} A_{4-5}^{coil}=\frac{\mu NI}{2(r_{2}-r_{1})(z_{2}-z_{1})}D_{ji}^{-1}g_{i}^{-1}X_{1}^{\prime }(g_{i}r)[\frac{1}{g_{j}^{3}}\int_{g_{j}r_{1}}^{g_{j}r_{2}}g_{j}r_{0}X_{1}^{\prime }(g_{j}r_{0})d(g_{j}r_{0})]\\ \cdot \{(e^{-gz}C_{510}-e^{gz}B_{510})\frac{\left(\beta_{1}+\beta_{3}-\lambda_{1}-\lambda_{3})(e^{g_{i}z_{2}}-e^{g_{i}z_{1}})+(\beta_{2}+\beta_{4}-\lambda_{2}-\lambda_{4})(e^{-g_{i}z_{1}}-e^{-g_{i}z_{2}}\right)}{(\lambda_{1}+\lambda_{3}-\beta_{1}-\beta_{3})C_{510}+(\lambda_{2}+\lambda_{4}-\beta_{2}-\beta_{4})B_{510}}+[2-e^{g_{i}(z_{1}-z)}-e^{g_{i}(z-z_{1})}]\} \end{array}$$

As illustrated in Figure 2b, the impedance of the multi-turn coil can be evaluated via the formula given below:

(57) $$\begin{array}{l} Z=\frac{j\omega 2\pi N}{I(z_{2}-z_{1})(r_{2}-r_{1})}\int_{r_{1}}^{r_{2}}\int_{z_{1}}^{z_{2}}rA_{4-5}^{coil}(r,z)drdz\\ =\frac{j\omega \mu \pi N^{2}}{{\left(r_{2}-r_{1}\right)}^{2}{\left(z_{2}-z_{1}\right)}^{2}}g^{-4}\chi_{1}(gr_{1},gr_{2})\cdot [2g(z_{2}-z_{1})+e^{g(z_{1}-z_{2})}-e^{g(z_{2}-z_{1})}+W_{1}W_{2}^{-1}W_{3}]D^{-1}g^{-3}\chi_{2}(gr_{1},gr_{2}) \end{array}$$

where the full definitions of all symbolic variables and auxiliary parameters are defined in Appendix A.

The derived closed form impedance formula describes the electromagnetic response of the probe and are numerically implemented and validated in Section 3. The computed impedance results are then compared against 2D/3D FEM benchmarks for model validation, and further utilised in Section 4 for parametric analysis of magnetic-core geometric influences on probe detection performance.

## 3. Numerical Analysis

Based on the TREE method, Equation (57) is derived to characterise the coil impedance of a composite-core probe situated above a multi-layered conductive specimen with embedded defects. The coil impedance can be evaluated numerically using custom scripts programmed in Mathematica 10 or MATLAB 7.0. The impedance calculation workflow primarily involves the computation of matrices such as **T**, **U**, **F**, **G**, **H**, **D**, and solving for the corresponding eigenvalues *q_i_*, *p_i_*, *m_i_*, and *g_i_*. All matrix entries are directly calculated according to the analytical expressions provided in Appendix A.

For eigenvalue solving, positive real eigenvalues *q_i_* are obtained by extracting the positive real roots of Equation (1) using Mathematica’s native BesselJzero function. For Equations (10), (20), and (32), only positive real roots are selected as valid eigenvalues *m_i_*, *p_i_*, and *g_i_*, which can be solved via numerical root-finding routines such as Mathematica’s NSolve [27]. As an alternative, eigenvalues *u_i_* can be computed from Equation (37) using the Newton–Raphson iterative method [28,29,30]. When root-finding solvers risk missing real roots due to closely spaced or near-degenerate eigenvalues, infinitesimal perturbations are applied to the input function to capture all real roots [31,32].

For impedance computations of the coil geometry shown in Figure 2b, all geometric and electromagnetic parameters of the T-core, II-core, excitation winding, and defective multi-layer conductor are specified in Table 1.

The geometric and electromagnetic parameters listed in Table 1 are selected as follows. Several geometrical dimensions are derived from typical real-world ferrite-core eddy current probe dimensions reported in the existing literature, while other dimensional values are adjusted to satisfy the subdomain division constraints of the present multi-subdomain T-II composite-core analytical model. Electromagnetic material parameters including relative permeability of ferrite cores and electrical conductivity of multi-layer conductors adopt typical datasheet values for commercial ferrite and common non-ferromagnetic conductive structural alloys.

Under a 1 kHz excitation condition, for the T-II composite magnetic core probe positioned above a multi-layer conductor containing a subsurface defect, the impedance calculated via high-precision 3D FEM simulation is adopted as the reference solution. By comparing the analytical model results obtained with different numbers of terms (*N*_s_) against this reference solution, the relative error and convergence characteristics of the model are quantitatively analysed.

The relative error between analytical predictions and 3D-FEM reference results is calculated as:

(58) $$E_{r}=\frac{\left|Z_{anal}-Z_{FEM}\right|}{\left|Z_{FEM}\right|}\times 100\%$$

where *Z*_anal_ denotes the impedance obtained from the proposed T-II composite-core analytical model, and *Z*_FEM_ is the reference impedance computed by 3D FEM simulation

As illustrated in Table 2, the resistance error reaches its minimum value at *N*_s_ = 30, whereas the reactance error keeps decreasing with increasing truncation-order *N*_s_. A further rise in *N*_s_ beyond 35 yields only marginal improvement in reactance accuracy but introduces noticeable growth in resistance deviation, together with a substantial increase in computational runtime. Considering the trade-off between overall calculation accuracy and computational burden, *N*_s_ = 35 is selected as the truncation order for all subsequent analytical calculations.

Across all numerical evaluations, an optimal balance between computational speed and numerical precision is achieved by setting the solution-domain truncation parameter *b* to 90 mm—equivalent to 11.3 times the coil outer radius *r*_2_—and fixing the series truncation order *N*_s_ at 35. Convergence tests were carried out in advance to confirm that further increasing *b* or *N*_s_ brings negligible improvement in numerical accuracy but significantly raises computation cost.

The normalised impedance variations in the composite-core probe, induced by multi-layer conductors with subsurface defects across a range of excitation frequencies, are computed and then benchmarked against those obtained from standalone T-core, II-core, and air-core probes. First, the coil complex impedance *Z* = *R* + j*X* is derived for the probe positioned above a multi-layer conductor with a buried defect. Two reference impedance states are subsequently solved for comparative analysis: *Z*_1_ = *R*_1_ + j*X*_1_—impedance of the probe suspended in free air; *Z*_2_ = *R*_2_ + j*X*_2_—impedance of the probe placed atop a multi-layer conductor of identical material and thickness, but defect-free.

The relative-permeability values *μ*_f1_ = 800 and *μ*_f2_ = 400 adopted for the T-core and II-core are typical initial permeability parameters taken from datasheets of two different commercial ferrite grades. It should be noted that the permeability of actual ferrite materials is strongly dependent on material grade, heat-treatment procedure, and manufacturing batch, and spatial heterogeneity of magnetic properties may also exist within practical ferrite components. In this work, all numerical cases use 1 A low-amplitude sinusoidal excitation within a 100 Hz–10 kHz frequency range. According to the FEM field monitoring results, the magnetic field amplitude inside both ferrite cores remains far below the saturation magnetic field threshold of conventional ferrites under such weak field conditions. Therefore, the ferrite material operates within its linear permeability region for all calculation examples presented in this manuscript.

The excitation frequency range of 0.1–10 kHz and lift-off distance of 0.1 mm adopted in this work are selected with practical inspection requirements taken into consideration. Within 0.1–10 kHz, the eddy current skin depth can cover the thickness range of the multi-layer conductive specimen and the burial depth of subsurface defects. This frequency band also avoids excessive deterioration of ferrite-core magnetic performance caused by ultra-high-frequency effects. The 0.1 mm lift-off represents a typical small gap condition in laboratory grade near field eddy current testing for multi-layer structural components. It should be noted that all numerical investigations reported here are constrained to this set of baseline conditions.

The coil reactance *X*_0_ for the cored probe in empty air (no conductive workpiece present) is also calculated.

The resistance and reactance perturbations arising purely from the multi-layered conductive substrate are defined as Δ*R*_1_ = *R* − *R*_1_ and Δ*X*_1_ = *X* − *X*_1_. In contrast, resistance and reactance deviations induced exclusively by subsurface defects within the layered conductors are given by Δ*R*_2_ = *R* − *R*_2_ and Δ*X*_2_ = *X* − *X*_2_. The normalised impedance variation is obtained by dividing the aforementioned impedance perturbations by the reactance X_0_ of the identical composite-core probe operated in free space with no conductive samples nearby.

**Figure 3:**
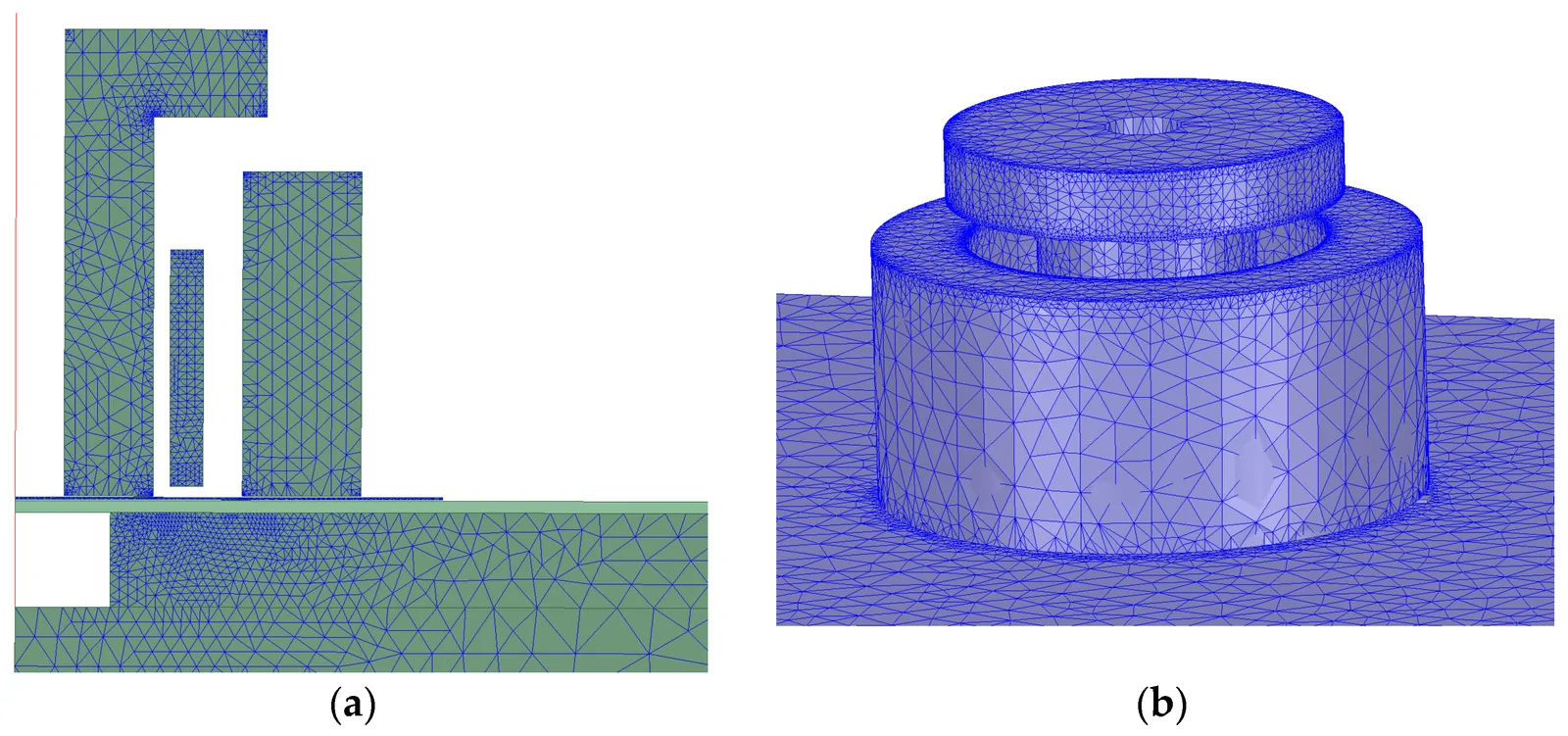
Numerical models built in ANSYS Maxwell: (**a**) 2D axisymmetric model; (**b**) full 3D model. The blue lines represent finite element meshes, and green areas denote solid components of the probe and specimen.

In parallel, the Finite Element Method (FEM) model shown in Figure 3 is established in ANSYS Maxwell 2021 R2 for cross-verification. Given the axisymmetric geometry of the probe and layered conductive specimen, the 2D model shown in Figure 3a is first established. Only half of the 2D cross-section needs to be modelled, which substantially reduces computation time. Additionally, the corresponding 3D model displayed in Figure 3b is also built to compare the differences in calculation accuracy and runtime between the 2D and 3D configurations. All dimensional and material parameters configured in the FEM model strictly conform to the specifications tabulated in Table 1.

Triangular finite elements are adopted to discretise the entire axisymmetric computational domain. Local mesh refinement is applied to regions with steep electromagnetic field gradients—mainly the area surrounding the excitation coil and subsurface embedded defects inside the specimen—to ensure high precision and stable convergence of simulation outputs.

For both 2D axisymmetric and full 3D models, the eddy current (magnetic) solver was utilised. Zero magnetic flux Neumann boundary conditions were imposed on the outer far field boundaries of the computational domain to mimic open air infinite space. The excitation coil was defined as a stranded winding with 400 turns driven by 1 A sinusoidal alternating current over the frequency range of 100 Hz–10 kHz.

Subsequently, the impedance predictions derived from the proposed TREE analytical framework are validated against the FEM simulation results. The FEM data act as reliable benchmark references to verify and validate the computational accuracy of the presented analytical method.

## 4. Results and Discussion

### 4.1. Model Accuracy Verification

This section utilises the analytical model derived in the preceding sections for the composite-core probe to investigate the frequency-dependent characteristics of its normalised impedance variation when placed above multi-layer conductive specimens with subsurface embedded defects. The impedance responses of the proposed composite-core probe are compared against those of conventional T-core, II-core, and air-core probes, and FEM simulations are utilised to validate the reliability and precision of the analytical predictions.

**Figure 4:**
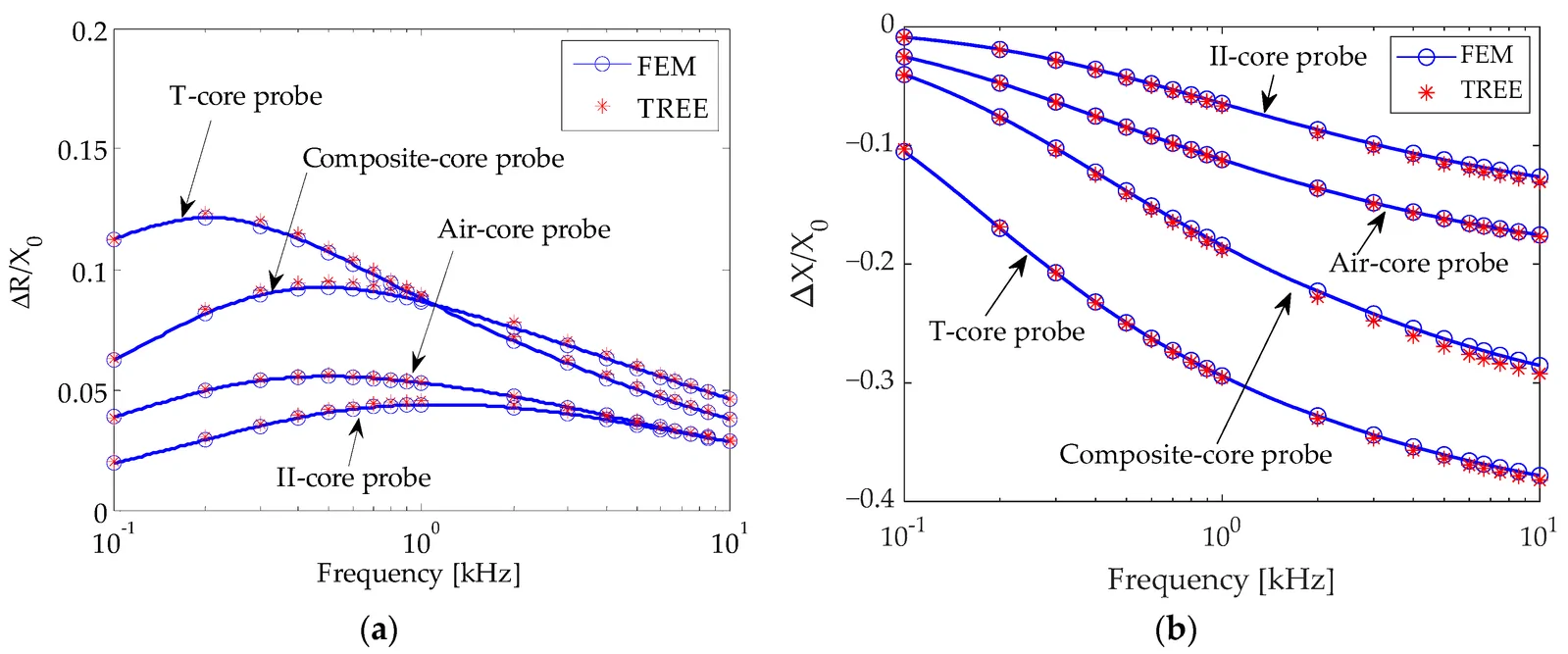
Normalised impedance variations in four probe configurations above defective multi-layer conductive specimens across variable excitation frequencies ΔR/X_0_: (**a**) normalised resistance variation; (**b**) normalised reactance variation ΔX/X_0_.

Figure 4a plots the normalised resistance variation ΔR/X_0_ caused by defective multi-layer conductors for all four probe designs as a function of excitation frequency. In the low-frequency regime below 1 kHz, the normalised resistance variation in the T-core probe exhibits a significantly higher magnitude than that of the composite-core probe. As the frequency rises into the medium-to-high frequency range above 1 kHz, the normalised resistance variation of the composite-core probe surpasses that of the T-core counterpart. Across the entire tested frequency band, the single II-core probe consistently exhibits the smallest normalised resistance variation among the four configurations, with values even lower than those of the air-core coil, due to its large distance from the coil.

Figure 4b illustrates the normalised reactance variation ΔX/X_0_ of the four probe coils generated by the defective multi-layer conductor versus excitation frequency. Over the full measured frequency spectrum, the T-core probe yields the largest absolute normalised reactance variation, followed by the composite-core probe. In contrast, the II-core probe delivers the weakest absolute normalised reactance variation, which remains lower than that of the air-core coil across the entire frequency range. As indicated in Figure 4, the analytical predictions achieve excellent consistency with the FEM simulation data under shared idealised assumptions. This cross check verifies the correct numerical implementation of the analytical derivation framework. As noted in the Abstract, this constitutes numerical verification rather than physical experimental validation against real-world probe hardware.

Table 3 quantitatively compares the conductor-induced coil impedance variations, corresponding computation time, and calculation accuracy obtained via the proposed analytical model and the FEM. The comparison is carried out on the composite-core probe shown in Figure 2b, which is placed above a defective multi-layer conductive specimen. All geometric and electromagnetic parameters of the probe and conductor are consistent with those listed in Table 1, and the excitation frequency is fixed at 1 kHz. Two distinct FEM numerical schemes are established in this work: a 2D axisymmetric model and a full 3D model. The simulation outputs from the 3D FEM model are treated as benchmark reference values to evaluate the relative errors of the 2D FEM results and analytical solutions.

The computational runtime tabulated herein refers to the total time required to calculate the conductor induced impedance variation, which covers two separate calculation cases: the probe positioned above the multi-layer conductor and the probe suspended in air. Uniform solver convergence settings are adopted for all FEM simulations: the maximum number of adaptive passes is set to 10, the relative convergence tolerance was set to 0.1%, and the nonlinear residual threshold was fixed at 0.0001. All analytical computations and FEM simulations are performed on a desktop computer equipped with an Intel G5400 (3.7 GHz) CPU and 32 GB RAM.

As illustrated by the data in Table 3, the runtime for one complete calculation of coil impedance variation varies drastically across the three approaches. The proposed analytical model consumes merely 9.94 s, while the 2D FEM takes 32 s, and the full 3D FEM requires the longest computation time of 1226 s. This evident disparity highlights the outstanding computational efficiency of the presented analytical framework. Furthermore, taking the 3D FEM results as benchmark reference values, the relative errors of the resistance and reactance components calculated by the analytical model are both below 2%.

All comparative evaluations are performed using the axisymmetric annular buried hole benchmark defect. Therefore, the conclusions cannot be directly generalised to arbitrary non-axisymmetric local defects. In addition, all results are obtained under perfect probe specimen alignment; the influence of probe offset, tilt, and lift-off variation will be studied in future work. A full discussion of model limitations is provided in Section 4.5.

From the perspective of reported accuracy in the existing eddy current modelling literature, TREE-based analytical models for ferrite-cored probes commonly yield relative errors ranging from 2% to 4% when validated against high fidelity FEM benchmarks. Against this background, the maximum relative error below 2% achieved in the present work demonstrates competitive prediction accuracy, especially considering the high complexity originating from the multi-subdomain coupling of the T-II composite-core geometry.

### 4.2. Frequency-Dependent Impedance of Equivalent Composite and Monolithic E-Core Probes

**Figure 5:**
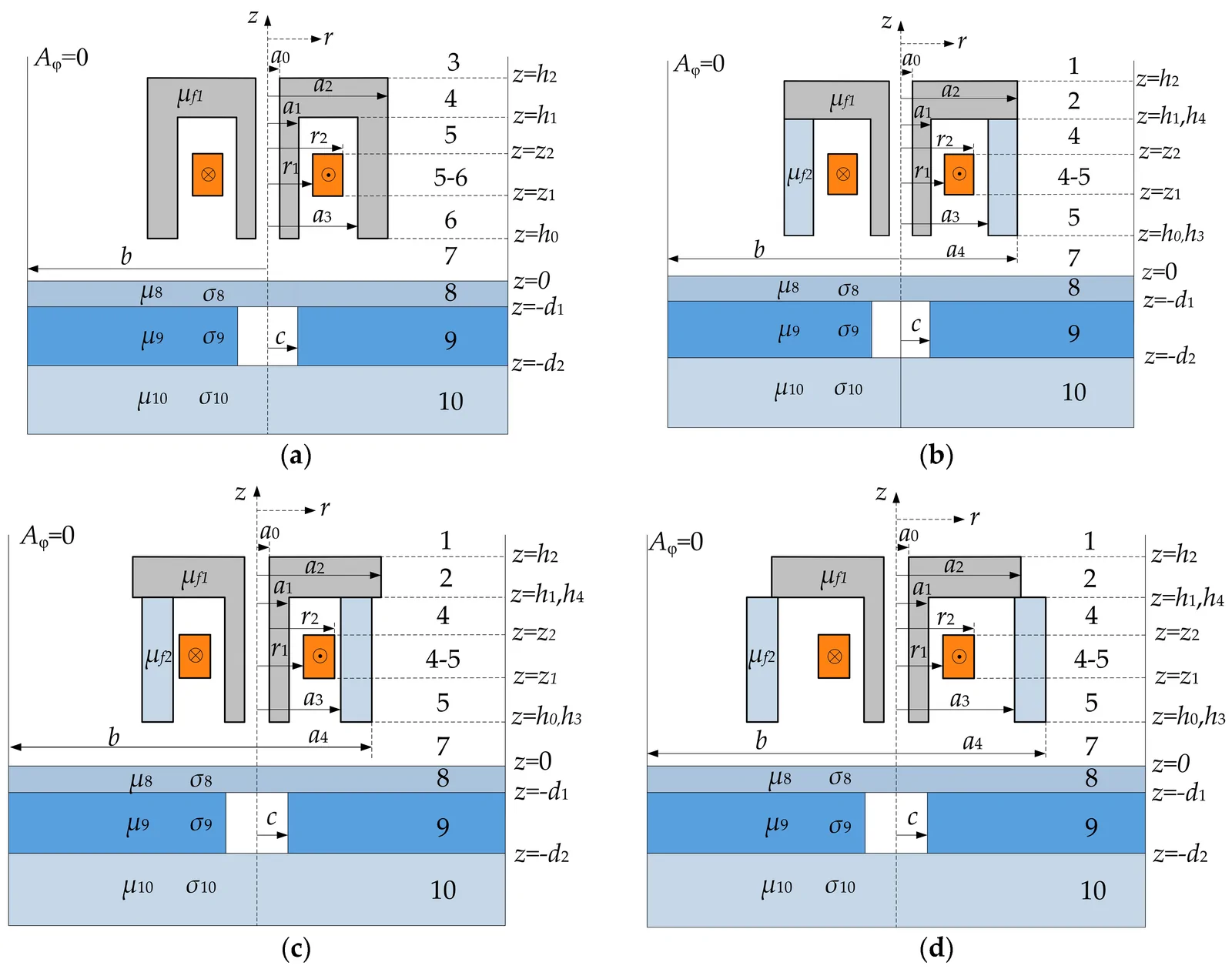
Four probe configurations arranged above defective multi-layer conductive specimens with subsurface embedded defects: (**a**) monolithic E-core probe; (**b**) dimensionally adjusted composite-core probe geometrically equivalent to the E-core in subfigure (**a**); (**c**) composite-core probe with reduced inner and outer radii of the II-core; (**d**) composite-core probe with enlarged inner and outer radii of the II-core. Colours distinguish different material regions; the orange block denotes the excitation coil, and ⊗/⊙ symbols indicate the direction of the excitation current.

In the preceding sections, an analytical model for the composite-core probe illustrated in Figure 2b was established. A distinctive feature of this design is that adjusting the geometric dimensions of the T-core and II-core allows them to be configured into a variety of geometric configurations, as presented in Figure 5b–d. When the outer radius *a*_2_ of the T-core is extended to satisfy *a*_2_ = *a*_4_, and the axial dimensions *h*_3_ and *h*_4_ of the II-core are stretched to meet *h*_3_ = *h*_0_ and *h*_4_ = *h*_1_, the two magnetic cores form the geometry shown in Figure 5b, which is geometrically identical to the standalone E-core structure in Figure 5a. Nevertheless, the similarities and differences in subsurface defect detection performance between this assembled E-core and the monolithic single E-core remain to be investigated. On the basis of the Figure 5b geometry, two additional composite-core layouts can be derived. By retaining the full T-core geometry and the fixed thickness of the II-core while reducing the inner and outer radii of the II-core, the probe structure in Figure 5c is obtained; conversely, expanding the inner and outer radii of the II-core yields the configuration in Figure 5d. These two structures deviate from the standard E-core design, which necessitates an evaluation of their flaw detection capabilities. This section takes the original composite-core probe (Figure 2b) positioned above a defective multi-layer conductive specimen as the research object. The dimensions of the T-core and II-core are first expanded to achieve full contact between the two magnetic cores. Then, with the II-core thickness kept constant, its inner and outer radii are modified to generate multiple composite-core configurations. Comparative analytical calculations and FEM simulations are carried out for all these probe structures, to explore how various composite magnetic core configurations influence defect detection performance under identical coil parameters.

**Figure 6:**
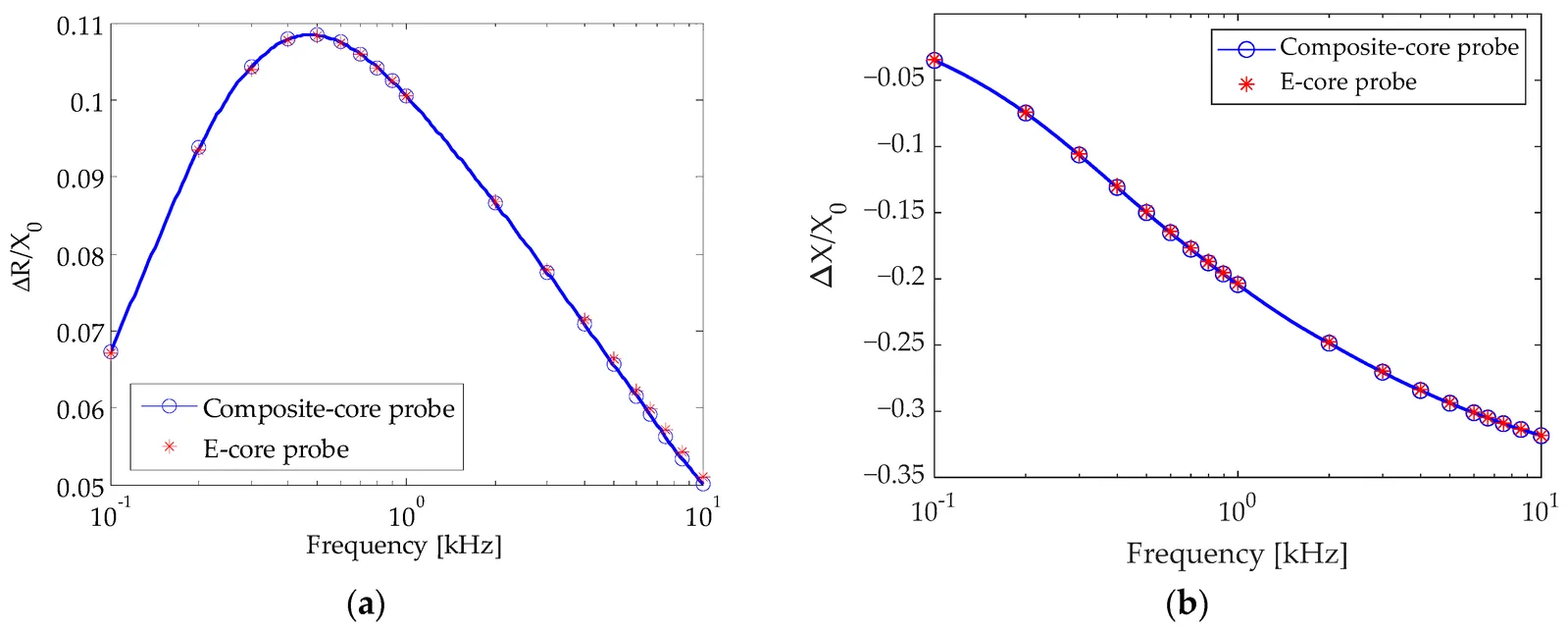
Normalised analytical impedance variations induced by defective multi-layer conductive specimens for composite-core and monolithic E-core probes with identical geometric and material parameters across varying excitation frequencies: (**a**) real component ΔR/X_0_; (**b**) imaginary component ΔX/X_0_.

Figure 6 plots the normalised impedance variations (real component ΔR/X_0_ and imaginary component ΔX/X_0_) of the two ECT probes in Figure 5a (monolithic E-core probe) and Figure 5b (equivalent composite-core probe) caused by defective multi-layer conductive specimens across the frequency range from 100 Hz to 10 kHz. Only analytical results are presented here to directly compare the impedance responses of the two identical-size magnetic core structures.

In Figure 6a, the normalised real impedance ΔR/X_0_ of both probes peaks near 0.5 kHz and then decreases monotonically with rising frequency. The composite-core probe maintains a slightly higher ΔR/X_0_ across the full frequency band, which indicates stronger eddy current excitation and larger resistive loss within the conductive specimen.

In Figure 6b, the normalised imaginary impedance ΔX/X_0_ of both structures declines monotonically as frequency increases. This trend arises because high-frequency eddy currents are confined to a thinner surface layer of the conductor due to the skin effect, which reduces the magnetic energy stored inside the conductive specimen. The monolithic E-core probe yields a slightly larger magnitude of ΔX/X_0_, reflecting a more prominent inductive response.

Overall, both probes generate measurable impedance variations when placed above defective layered conductors. The composite-core probe produces a more significant real part response, while the imaginary impedance curves of the two structures show nearly perfect overlap. This demonstrates that the composite-core probe shares almost identical frequency response characteristics with the monolithic E-core probe under matched dimensional and material settings.

It should be emphasised that the nearly overlapping impedance curves only reflect similar external electromagnetic responses under the ideal geometric assumption used in this simulation, rather than complete physical equivalence between the assembled composite-core and monolithic E-core probes. For the dimension-stretched assembled T-II composite-core configuration, an interfacial contact surface exists between the T-core and II-core. In practical hardware fabrication, this splicing interface inevitably introduces additional air-gap magnetic reluctance and extra magnetic circuit losses, which are absent in the integrated monolithic E-core without internal splicing interfaces. In the numerical model presented, perfect intimate contact with zero physical clearance is assumed at the splicing boundary, and the magnetic reluctance originating from tiny manufacturing gaps is not incorporated. If finite machining-induced air gaps occur at the contact interface in real-world probes, non-negligible discrepancies in impedance, magnetic loss, and internal magnetic circuit distribution will appear between these two structures. Therefore, approximate consistency of external impedance cannot be treated as evidence that their internal magnetic circuit behaviours are identical.

### 4.3. Effect of II-Core Radius on Eddy Current and Magnetic Flux Distribution

**Figure 7:**
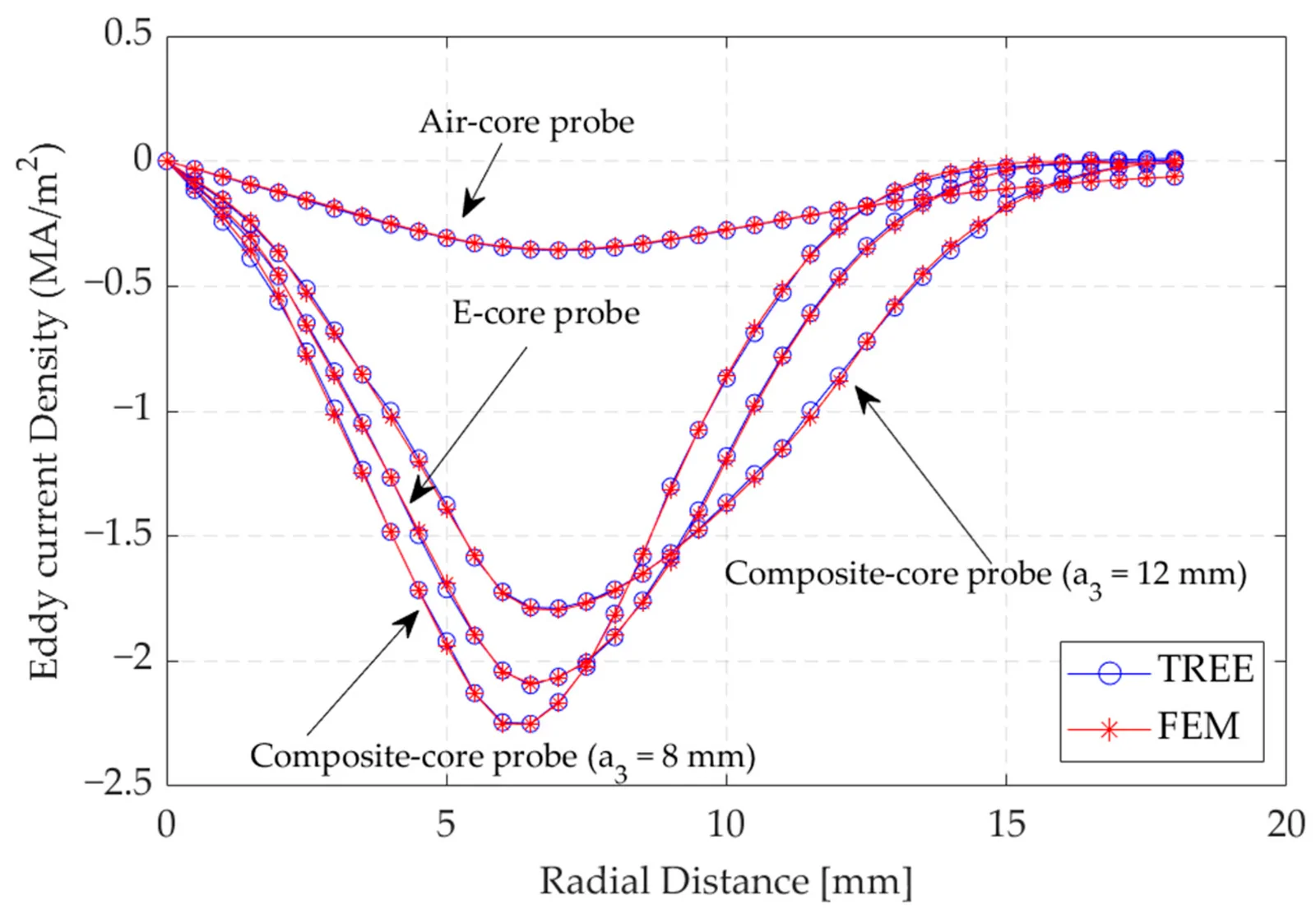
Eddy current density at 0.2 mm subsurface depth of the multi-layer conductive specimen versus radial distance from the *Z*-axis under 1 kHz, 1 A excitation.

Figure 7 compares the eddy current density distribution at a depth of 0.2 mm beneath the specimen surface as a function of radial distance from the probe central axis. All tests adopt fixed excitation conditions: 1 kHz excitation frequency and 1 A driving current. Eddy current density data are obtained separately via the proposed TREE analytical framework and FEM simulations for cross-verification. Four probe configurations, all with an identical lift-off height, are analysed: monolithic E-core probe (Figure 5a); composite-core probe with *a*_3_ = 8 mm and *a*_4_ = 13 mm (Figure 5c); composite-core probe with *a*_3_ = 12 mm and *a*_4_ = 17 mm (Figure 5d); and the air-core probe.

As illustrated in Figure 7, the air-core probe generates far weaker eddy currents than the three magnetic-core probes under identical excitation. Within a radial distance below 7.5 mm, the eddy current magnitude of the monolithic E-core probe exceeds that of the large-radius composite-core probe (*a*_3_ = 12 mm, *a*_4_ = 17 mm). However, its eddy current density remains lower than that of the small-radius composite-core probe (*a*_3_ = 8 mm, *a*_4_ = 13 mm). When the radial distance exceeds 9 mm, this magnitude relationship reverses: the monolithic E-core probe produces stronger eddy currents than the small-radius composite-core probe but weaker eddy currents than the large-radius composite-core probe.

This observation demonstrates that adjusting the inner and outer radii of the II-core allows the composite-core probe to achieve higher eddy current density magnitudes over the entire radial range compared with the monolithic E-core probe. This unique feature can be exploited to improve subsurface defect detection sensitivity and expand the effective detection coverage of the probe.

Magnetic cores concentrate magnetic flux to generate much higher eddy current density than air-core coils, and the TREE analytical curves match the FEM results well in Figure 7.

**Figure 8:**
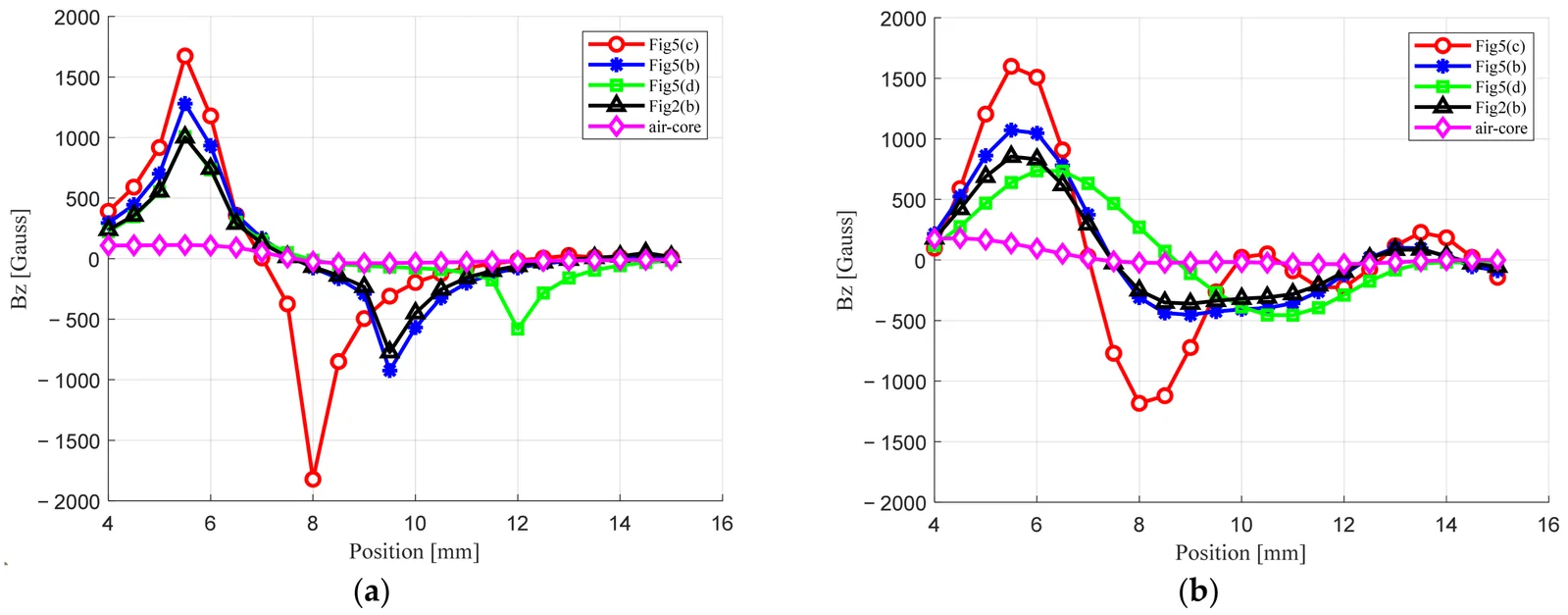
Radial distribution of vertical magnetic flux density *B_z_* at 0.1 mm lift-off above the multi-layer conductive specimen: (**a**) FEM simulation results; (**b**) analytical calculation results.

Figure 8 compares the vertical magnetic flux density *B*_z_ along a radial sampling line ranging from 4 mm to 15 mm (0.5 mm sampling step) at a lift-off height of 0.1 mm above defective multi-layer conductive specimens. The comparison covers three composite-core configurations (Figure 5b–d) and an air-core coil under 1 kHz, 1 A excitation. Figure 8a presents FEM simulation results, and Figure 8b corresponds to the analytical solutions from the TREE model.

Minor amplitude discrepancies between analytical and FEM results stem from geometric simplifications in modelling and inherent numerical truncation errors, yet all *B_z_* curves share identical overall trends. Specifically, the peak/valley positions, fluctuation profiles, and transition intervals of the flux density curves achieve excellent consistency.

Under identical excitation, all ferrite-core probes generate far larger vertical flux density *B_z_* at 0.1 mm lift-off than the air-core coil. A prominent *B_z_* peak appears near *r* = 5.7 mm, corresponding to the outer radius of the T-core central column; a secondary flux extremum occurs near the inner radius of the II-core. Among all composite-core designs, the structure in Figure 5c delivers the maximum *B_z_* magnitude at both extrema. Its outer extremum is located at approximately *r* = 8 mm, which exactly coincides with its II-core inner radius *a*_3_ = 8 mm.

The second strongest *B_z_* response is observed for the equivalent E-core composite structure in Figure 5b. This geometry is formed by axially extending the T-core and II-core to achieve full contact while retaining the original II-core radii; its II-core inner radius *a*_3_ = 9.55 mm matches the position of the secondary flux peak. This confirms that positioning the II-core closer via axial extension to place the magnetic core closer to the excitation coil produces stronger magnetic field coupling than the conventional monolithic E-core probe.

The structures in Figure 5d and Figure 2b exhibit weaker *B_z_* amplitudes. Near the central axis, their peak flux magnitudes are comparable. For the outboard extremum far from the axis, the peak position of the original composite-core probe (Figure 2b) appears around *r* = 9.55 mm, consistent with its II-core inner radius. By contrast, the II-core radii of the design in Figure 5d increase to *a*_3_ = 12 mm, shifting its corresponding flux extremum to *r* = 12 mm.

Despite forming a closed magnetic circuit with fully contacted T-core and II-core, the larger II-core radii in Figure 5d place the magnetic core farther from the coil, leading to overall flux performance similar to the non-contact composite-core probe in Figure 2b. Consequently, this closed-core design offers a distinct advantage: it shifts the peak magnetic excitation field to a position farther from the coil central axis.

### 4.4. Mechanism Analysis of Composite Magnetic Core Flux Focusing

Since different magnetic core structures produce varying *B_z_* magnitudes in the lift-off air gap, they correspondingly induce eddy currents of different intensities at the 0.2 mm subsurface layer of the specimen. To reveal the underlying physical mechanism of eddy current distribution, the air-gap vertical flux density *B_z_* at 0.1 mm lift-off is further analysed.

As shown in Figure 8, the proposed composite magnetic core reduces the magnetic reluctance of the air gap and concentrates magnetic flux in the lift-off region adjacent to the specimen surface, yielding much higher *B_z_* magnitude than the air-core probe.

The intensified magnetic field coupling within the lift-off gap directly accounts for the elevated eddy current density observed at the 0.2 mm subsurface layer (discussed in the previous section). These results verify that the composite-core effectively concentrates magnetic flux and enhances the excitation performance of the eddy current probe.

### 4.5. Limitations of This Work

Although the proposed TREE-based analytical model demonstrates high consistency with FEM predictions and computational efficiency, several inherent limitations exist due to theoretical simplifications and validation constraints:

1. Idealised Modelling Assumptions: The model relies on axisymmetric assumptions for both the probe and the defect (annular hole). Consequently, it cannot directly address arbitrary non-axisymmetric flaws (e.g., inclined cracks) or complex geometries like curved components and gradient-thickness layers. Additionally, the ferrite cores are assumed to be linear and homogeneous, neglecting practical nonlinear saturation effects and spatial heterogeneity. Interlayer interfaces are also idealised as perfectly smooth and tight, ignoring flux attenuation caused by roughness or air gaps.

2. Limited Validation Scope: The study is currently restricted to the 0.1–10 kHz frequency band with a fixed probe alignment. The model’s robustness under probe tilting, lateral offset, and ultra-high frequencies remains to be verified. Furthermore, validation relies solely on FEM cross-verification; the lack of physical prototype experiments means the analytical predictions have not yet been calibrated against measured impedance data.

3. Forward Problem Focus: This work addresses the forward problem of calculating impedance responses rather than the inverse problem of quantitative defect reconstruction. While the model effectively evaluates global impedance perturbations, it does not derive explicit quantitative relationships between defect parameters (size, depth) and impedance variations, nor does it resolve microscopic field distortions at defect edges.

4. Material Constraints: The derivation is strictly valid for non-ferromagnetic multi-layer conductors. Complex magnetic coupling effects associated with ferromagnetic substrates are not included in the current framework.

## 5. Conclusions

This paper presents a novel analytical impedance model for a composite T-II core ECT probe inspecting defective multi-layer conductive specimens. Based on the TREE method, the model provides explicit, computationally efficient impedance expressions that show excellent agreement with ANSYS Maxwell FEM simulations while significantly reducing calculation time.

Comparative analysis demonstrates that the proposed composite-core design achieves superior magnetic flux focusing and detection sensitivity for hidden subsurface defects compared to single T-core, II-core, and monolithic E-core probes. By offering flexible structural reconfiguration and a robust theoretical foundation, this work supports applications in computer-aided simulation, high-sensitivity sensor optimisation, and embedded calculation for portable instruments.

Future research will focus on the following directions:

1. Experimental Verification: Physical composite-core prototypes will be fabricated to validate the model’s prediction performance and detection sensitivity in real-world scenarios, moving beyond ideal numerical verification.

2. Model Generalisation: The analytical framework will be extended to address non-axisymmetric defects, complex geometries (e.g., curvature, thickness gradients), and interlayer air gaps. Additionally, non-linear magnetic properties will be incorporated to overcome current weak-field limitations.

3. Quantitative Inversion: Future work will establish parametric mapping between defect geometry and impedance responses to support quantitative inversion. Broader parametric studies covering diverse frequencies, lift-offs, and probe misalignments will be conducted to enhance design robustness.

## Figures and Tables

**Table 1:** Geometric and electromagnetic parameters of the magnetic core, excitation coil, and defective multi-layer conductive specimen for analytical calculations and finite element simulations.

Category	Parameter Description	Symbol	Value	Unit
T-core	Air gap radius	*a*_0_	2.05	mm
	Inner core radius	*a*_1_	5.8	mm
	Outer core radius	*a*_2_	10.55	mm
	Inner core height	*h*_1_	16.2	mm
	Outer core height	*h*_2_	19.9	mm
	Core lift-off height	*h*_0_	0.2	mm
	Relative permeability	*μ_f_*_1_	800	—
II-core	Inner core radius	*a*_3_	9.55	mm
	Outer core radius	*a*_4_	14.55	mm
	Core height	*h*_4_	13.9	mm
	Core lift-off height	*h*_3_	0.2	mm
	Relative permeability	*μ_f_*_2_	400	—
Excitation coil	Inner coil radius	*r*_1_	6.5	mm
	Outer coil radius	*r*_2_	7.9	mm
	Vertical offset	*z*_1_	0.6	mm
	Vertical distance	*z*_2_	10.6	mm
	Number of turns	*N*	400	—
Multi-layer Conductor	Central hole radius	*c*	4	mm
	Height of first layer	*d*_1_	0.5	mm
	Height of second layer	*d*_2_	4.5	mm
	Truncation domain radius	*b*	90	mm
	Conductivity	*σ*_8_, *σ*_9_	36	MS⋅m^−1^
	Conductivity	*σ*_10_	15	MS⋅m^−1^
	Relative permeability	*μ*_8_, *μ*_9_, *μ*_10_	1	—

**Table 2:** Convergence analysis of computed coil impedance for varying TREE truncation order *N*_s_; fixed truncation radius *b* = 90 mm, and excitation frequency *f* = 1 kHz.

*N*_s_	Impedance Change (Ω)		Relative Error (%)	
	Resistance	Reactance	Resistance	Reactance
3D FEM	5.9785	−12.7731	—	—
10	5.7082	−13.9099	4.5212	8.9
20	5.9547	−13.6106	0.3981	6.5567
30	5.9691	−12.6873	0.1572	0.6717
35	6.0927	−12.8526	1.9102	0.6224
40	6.1132	−12.8445	2.2531	0.559
45	6.2515	−12.8083	4.5664	0.2756

**Table 3:** Comparison of conductor-induced coil impedance variations, relative errors, and computational runtime at 1 kHz excitation frequency.

Model	Impedance Change (Ω)		Relative Error (%)		Runtime for a Single Computation (s)
	Resistance	Reactance	Resistance	Reactance	
TREE Analytical	6.0927	−12.8526	1.9102	0.6224	9.94
2D FEM	5.9837	−12.7831	0.087	0.079	32
3D FEM (Benchmark)	5.9785	−12.7731	—	—	1226

## Data Availability

The original contributions presented in this study are included in the article. Further inquiries can be directed to the corresponding author.

## Abbreviations

The following abbreviations are used in this manuscript:

TREE	Truncated Region Eigenfunction Expansion
ECT	Eddy current testing
NDT	Non-destructive testing
FEM	Finite Element Method

## Appendix A

The definitions of all symbols used in Equation (57) are listed below.

(A1) $$\chi_{1}(gr_{1},gr_{2})=\int_{gr_{1}}^{gr_{2}}(gr)X_{1}^{\prime }(gr)d(gr)$$

(A2) $$\chi_{2}(gr_{1},gr_{2})=\int_{gr_{1}}^{gr_{2}}(gr_{0})X_{1}^{\prime }(gr_{0})d(gr_{0})$$

(A3) $$W_{1}=(e^{-gz_{1}}-e^{-gz_{2}})C_{510}-(e^{gz_{2}}-e^{gz_{1}})B_{510}$$

(A4) $$W_{2}=(\lambda_{1}+\lambda_{3}-\beta_{1}-\beta_{3})C_{510}+(\lambda_{2}+\lambda_{4}-\beta_{2}-\beta_{4})B_{510}$$

(A5) $$W_{3}=(\beta_{1}+\beta_{3}-\lambda_{1}-\lambda_{3})(e^{gz_{2}}-e^{gz_{1}})+(\beta_{2}+\beta_{4}-\lambda_{2}-\lambda_{4})(e^{-gz_{1}}-e^{-gz_{2}})$$

(A6) $$\lambda_{1}=(T^{-1}-U^{-1})Fe^{m(h_{1}-h_{2})}F^{-1}(G+H)e^{p(h_{4}-h_{1})}(G^{*-1}+H^{*-1})De^{-gh_{4}}$$

(A7) $$\lambda_{2}=(T^{-1}-U^{-1})Fe^{m(h_{1}-h_{2})}F^{-1}(G+H)e^{p(h_{4}-h_{1})}(-G^{*-1}+H^{*-1})De^{gh_{4}}$$

(A8) $$\lambda_{3}=(T^{-1}-U^{-1})Fe^{m(h_{1}-h_{2})}F^{-1}(-G+H)e^{p(h_{1}-h_{4})}(-G^{*-1}+H^{*-1})De^{-gh_{4}}$$

(A9) $$\lambda_{4}=(T^{-1}-U^{-1})Fe^{m(h_{1}-h_{2})}F^{-1}(-G+H)e^{p(h_{1}-h_{4})}(G^{*-1}+H^{*-1})De^{gh_{4}}$$

(A10) $$\beta_{1}=(T^{-1}+U^{-1})Fe^{m(h_{2}-h_{1})}F^{-1}(-G+H)e^{p(h_{4}-h_{1})}(G^{*-1}+H^{*-1})De^{-gh_{4}}$$

(A11) $$\beta_{2}=(T^{-1}+U^{-1})Fe^{m(h_{2}-h_{1})}F^{-1}(-G+H)e^{p(h_{4}-h_{1})}(-G^{*-1}+H^{*-1})De^{gh_{4}}$$

(A12) $$\beta_{3}=(T^{-1}+U^{-1})Fe^{m(h_{2}-h_{1})}F^{-1}(G+H)e^{p(h_{1}-h_{4})}(-G^{*-1}+H^{*-1})De^{-gh_{4}}$$

(A13) $$\beta_{4}=(T^{-1}+U^{-1})Fe^{m(h_{2}-h_{1})}F^{-1}(G+H)e^{p(h_{1}-h_{4})}(G^{*-1}+H^{*-1})De^{gh_{4}}$$

(A14) $$C_{510}=\frac{1}{2}e^{gh_{3}}D^{-1}[(G^{*}+H^{*})e^{-ph_{3}}C_{610}+(-G^{*}+H^{*})e^{ph_{3}}B_{610}]$$

(A15) $$B_{510}=\frac{1}{2}e^{-gh_{3}}D^{-1}[(-G^{*}+H^{*})e^{-ph_{3}}C_{610}+(G^{*}+H^{*})e^{ph_{3}}B_{610}]$$

(A16) $$C_{610}=\frac{1}{2}e^{ph_{0}}F^{*-1}[(M+M^{*})e^{-qh_{0}}C_{710}+(-M+M^{*})e^{qh_{0}}B_{710}]$$

(A17) $$B_{610}=\frac{1}{2}e^{-ph_{0}}F^{*-1}[(-M+M^{*})e^{-qh_{0}}C_{710}+(M+M^{*})e^{qh_{0}}B_{710}]$$

(A18) $$C_{710}=\frac{1}{2}[(\mu_{8}^{-1}+qs_{8}^{-1})C_{810}+(\mu_{8}^{-1}-qs_{8}^{-1})B_{810}]$$

(A19) $$B_{710}=\frac{1}{2}[(\mu_{8}^{-1}-qs_{8}^{-1})C_{810}+(\mu_{8}^{-1}+qs_{8}^{-1})B_{810}]$$

(A20) $$C_{810}=\frac{1}{2}e^{-s_{8}d_{1}}[(s_{8}q^{-1}E^{-1}V+E^{-1}N)e^{ud_{1}}C_{910}+(-s_{8}q^{-1}E^{-1}V+E^{-1}N)e^{-ud_{1}}B_{910}]$$

(A21) $$B_{810}=\frac{1}{2}e^{s_{8}d_{1}}[(-s_{8}q^{-1}E^{-1}V+E^{-1}N)e^{ud_{1}}C_{910}+(s_{8}q^{-1}E^{-1}V+E^{-1}N)e^{-ud_{1}}B_{910}]$$

(A22) $$C_{910}=\frac{1}{2}e^{-ud_{2}}(-V^{-1}Eqs_{10}^{-1}+N^{-1}E)e^{-s_{10}d_{2}}$$

(A23) $$B_{910}=\frac{1}{2}e^{ud_{2}}(V^{-1}Eqs_{10}^{-1}+N^{-1}E)e^{-s_{10}d_{2}}$$

In the analytical model and calculations, matrices including **T**, **U**, **F**, **G**, **H**, **G***, **D**, **H***, **M**, **M***, and **N** are defined as follows:

(A24) $$T=\int_{0}^{a_{0}}rJ_{0}(mr)J_{0}(q^{T}r)+\frac{1}{\mu_{f1}}\int_{a_{0}}^{a_{2}}rR_{0}(mr)J_{0}(q^{T}r)+\int_{a_{2}}^{b}rR\prime_{0}(mr)J_{0}(q^{T}r)$$

(A25) $$U=\int_{0}^{a_{0}}rJ_{1}(mr)J_{1}(q^{T}r)+\int_{a_{0}}^{a_{2}}rR_{1}(mr)J_{1}(q^{T}r)+\int_{a_{2}}^{b}rR\prime_{1}(mr)J_{1}(q^{T}r)$$

(A26) $$F=\int_{0}^{a_{0}}rJ_{1}(mr)J_{1}(m^{T}r)dr+\frac{1}{\mu_{f1}}\int_{a_{0}}^{a_{2}}rR_{1}(mr)R_{1}(m^{T}r)dr+\int_{a_{2}}^{b}rR_{1}^{\prime }(mr)R_{1}^{\prime }(m^{T}r)dr$$

(A27) $$G=\int_{0}^{a_{0}}rJ_{0}(mr)J_{0}(p^{T}r)dr+\frac{1}{\mu_{f1}}\int_{a_{0}}^{a_{1}}rR_{0}(mr)L_{0}(p^{T}r)dr+\frac{1}{\mu_{f1}}\int_{a_{1}}^{a_{2}}rR_{0}(mr)L_{0}^{\prime }(p^{T}r)dr+\int_{a_{2}}^{b}rR_{0}^{\prime }(mr)L_{0}^{\prime }(p^{T}r)dr)$$

(A28) $$H=\int_{0}^{a_{0}}rJ_{1}(mr)J_{1}(p^{T}r)dr+\frac{1}{\mu_{f1}}\int_{a_{0}}^{a_{1}}rR_{1}(mr)L_{1}(p^{T}r)dr+\int_{a_{1}}^{a_{2}}rR_{1}(mr)L_{1}^{\prime }(p^{T}r)dr+\int_{a_{2}}^{b}rR_{1}^{\prime }(mr)L_{1}^{\prime }(p^{T}r)dr$$

(A29) $$\begin{array}{l} G*=\int_{0}^{a_{0}}rJ_{0}(gr)J_{0}(p^{T}r)+\frac{1}{\mu_{f1}}\int_{a_{0}}^{a_{1}}rX_{0}(gr)L_{0}(p^{T}r)dr)+\int_{a_{1}}^{a_{3}}rX_{0}^{\prime }(gr)L_{0}^{\prime }(p^{T}r)dr\\ +\frac{1}{\mu_{f2}}\int_{a_{3}}^{a_{4}}rX_{0}^{\prime \prime }(gr)L_{0}^{\prime }(p^{T}r)dr+\int_{a_{4}}^{b}rX_{0}^{\prime \prime \prime }(gr)L_{0}^{\prime }(p^{T}r)dr \end{array}$$

(A30) $$\begin{array}{l} D=\int_{0}^{a_{0}}rJ_{0}(gr)J_{0}(g^{T}r)dr+\frac{1}{\mu_{f1}}\int_{a_{0}}^{a_{1}}rX_{0}(gr)X_{0}(g^{T}r)dr+\int_{a_{1}}^{a_{3}}rX_{0}^{\prime }(gr)X_{0}^{\prime }(g^{T}r)dr\\ +\frac{1}{\mu_{f2}}\int_{a_{3}}^{a_{4}}rX_{0}^{\prime \prime }(gr)X_{0}^{\prime \prime }(g^{T}r)dr+\int_{a_{4}}^{b}rX_{0}^{\prime \prime \prime }(gr)X_{0}^{\prime \prime \prime }(g^{T}r)dr \end{array}$$

(A31) $$\begin{array}{l} H*=\int_{0}^{a_{0}}rJ_{1}(gr)J_{1}(p^{T}r)+\frac{1}{\mu_{f1}}\int_{a_{0}}^{a_{1}}rX_{1}(gr)L_{1}(p^{T}r)dr+\int_{a_{1}}^{a_{3}}rX_{1}^{\prime }(gr)L_{1}^{\prime }(p^{T}r)dr\\ +\int_{a_{3}}^{a_{4}}rX_{1}^{\prime \prime }(gr)L_{1}^{\prime }(p^{T})dr+\int_{a_{4}}^{b}rX_{1}^{\prime \prime \prime }(gr)L_{1}^{\prime }(p^{T}r)dr \end{array}$$

(A32) $$F^{*}=\int_{0}^{a_{0}}rJ_{0}(pr)J_{0}(p^{T}r)+\frac{1}{\mu_{f1}}\int_{a_{0}}^{a_{1}}rL_{0}(pr)L_{0}(p^{T}r)dr)+\int_{a_{1}}^{b}rL_{0}^{\prime }(pr)L_{0}^{\prime }(p^{T}r)dr$$

(A33) $$M=\int_{0}^{a_{0}}rJ_{0}(pr)J_{0}(q^{T}r)dr+\frac{1}{\mu_{f1}}\int_{a_{0}}^{a_{1}}rL_{0}(pr)J_{0}(q^{T}r)dr+\int_{a_{1}}^{b}rL_{0}^{\prime }(pr)J_{0}(q^{T}r)dr$$

(A34) $$M^{*}=\int_{0}^{a_{0}}rJ_{1}(pr)J_{1}(q^{T}r)dr+\int_{a_{0}}^{a_{1}}rL_{1}(pr)J_{1}(q^{T}r)dr+\int_{a_{1}}^{b}rL_{1}^{\prime }(pr)J_{1}(q^{T}r)dr$$

(A35) $$V=F_{1}(vc)\int_{0}^{c}J_{0}(ur)J_{0}(q^{T}r)dr+J_{1}(uc)v_{i}u_{i}^{-1}\int_{c}^{b}rF_{0}(vr)J_{0}(q^{T}r)dr$$

(A36) $$N=F_{1}(vc)\int_{0}^{c}rJ_{1}(ur)J_{1}(q^{T}r)dr+\mu_{9}^{-1}J_{1}(uc)\int_{c}^{b}rF_{1}(vr)J_{1}(q^{T}r)dr$$
